# HOSPITAL MANAGERS’ NEED FOR INFORMATION ON HEALTH TECHNOLOGY INVESTMENTS

**DOI:** 10.1017/S0266462315000665

**Published:** 2015

**Authors:** Anne Mette Ølholm, Kristian Kidholm, Mette Birk-Olsen, Janne Buck Christensen

**Affiliations:** Department for Quality, Research and HTA, Odense University Hospitalanne.mette.oelholm@rsyd.dk; Department for Quality, Research and HTA, Odense University Hospital

**Keywords:** Hospital, HTA, Review, Decision making, Criteria

## Abstract

**Objectives:** There is growing interest in implementing hospital-based health technology assessment (HB-HTA) as a tool to facilitate decision making based on a systematic and multidisciplinary assessment of evidence. However, the decision-making process, including the informational needs of hospital decision makers, is not well described. The objective was to review empirical studies analysing the information that hospital decision makers need when deciding about health technology (HT) investments.

**Methods:** A systematic review of empirical studies published in English or Danish from 2000 to 2012 was carried out. The literature was assessed by two reviewers working independently. The identified informational needs were assessed with regard to their agreement with the nine domains of EUnetHTA's Core Model.

**Results:** A total of 2,689 articles were identified and assessed. The review process resulted in 14 relevant studies containing 74 types of information that hospital decision makers found relevant. In addition to information covered by the Core Model, other types of information dealing with political and strategic aspects were identified. The most frequently mentioned types of information in the literature related to clinical, economic and political/strategic aspects. Legal, social, and ethical aspects were seldom considered most important.

**Conclusions:** Hospital decision makers are able to describe their information needs when deciding on HT investments. The different types of information were not of equal importance to hospital decision makers, however, and full agreement between EUnetHTA's Core Model and the hospital decision-makers’ informational needs was not observed. They also need information on political and strategic aspects not covered by the Core Model.

Hospitals are often the main entry level for new health technologies (HT), and they invest a considerable volume of resources in implementation of new HT. There is growing interest in hospital-based health technology assessment (HB-HTA) as a tool to facilitate hospital decision making that is based on a systematic and multidisciplinary assessment of the evidence for new HTs (1). The background knowledge and available scientific evidence that underpins HB-HTA is the same as that used for national and regional HTA. However, the information required for making decisions about the introduction of a new HT, the time frame and the relative importance given to the different types of information may differ according to the organizational level of the healthcare sector at which decisions are made (2).

HB-HTA links evidence-based clinical data with the unique organizational and economic implications of a new HT at the local hospital level, thus providing shorter and more timely HTA reports to hospital decision makers (2). The decision-making process at hospitals, including the informational needs of hospital and clinical managers when deciding whether or not to invest in health technologies, is generally not well described, however (3).

One of the most widely used set of guidelines on how to perform and report HTAs is the Core Model developed by EUnetHTA (the European network for Health Technology Assessment), from a collaboration of primarily national HTA institutions. The Core Model describes a large number of potential elements for assessment (topics and items) that are divided into nine domains (4). HB-HTA products should be aligned with the needs of the final decision makers at hospital level, but the level of agreement between the Core Model and the informational needs of hospital decision makers is currently unknown.

The primary objective of this study was to review empirical studies that analyze the information hospital decision makers want to have at their disposal before making decisions on HT investments. Furthermore, we wished to rank the importance of the requested information based on the number and frequency of mentions in the identified literature.

This systematic review is part of the European research project AdHopHTA (Adopting Hospital Based Health Technology Assessment in EU; http://www.adhophta.eu/) which aims to strengthen the use and impact of HTA in hospital settings. The results of this literature review will be used to develop qualitative interviews and a questionnaire survey among hospital decision makers in Europe.

## METHODS

### Identification of Information Types

A systematic review of empirical studies was carried out to identify the informational needs of hospital and clinical managers when deciding about HT investments. We searched for empirical studies published in English or Danish from 2000 to 2012 (November) in the PubMed, Embase, Cochrane Library, and Web of Science databases. The subject of the literature search had three topics (decision maker [who], informational need [what], hospital setting [where]) and we limited the search to specific study designs. For each of the three topics, we defined the various queries (with similar search terms using both Medical Subject Headings [MeSH] and free text) and combined them into a final query. The search strategy was reviewed and refined by a senior research librarian. Supplementary Table 1 provides the full search histories.

After exclusion of 517 duplicates between the databases, the 2,689 articles that were identified as being potentially relevant were reviewed by two authors (K.K. and A.M.O.), working independently of each other. In case of disagreement, a third opinion (M.B.O.) was sought and in- or exclusion was resolved by discussion among the authors. Assessment of the literature was carried out in two phases—first by examining relevance by reading only the title and abstract, and then by reading the full text of those articles still deemed relevant. A kappa coefficient was calculated to measure the correlation between assessments made by the two independent reviewers. This coefficient takes into account the fact that part of the observed correlation between two assessments is due to chance (5).

When reviewing the literature, we looked specifically for evidence of which information that hospital decision makers needed when deciding on HT investments. This information was retrieved from empirical studies of hospital managers’ attitudes and of decision-making processes in hospitals. Inclusion criteria were: (i) articles reporting on informational needs in a decision-making situation, (ii) in a hospital context, and (iii) based on an empirical study (not commentaries, letters, opinions, etc.). Systematic reviews of empirical studies were also accepted. The included articles were reviewed and the main types of information needed by hospital decision makers were listed.

### Categorization of Information Types

The different types of information requested by hospital decision makers were categorized according to the nine domains of EUnetHTA's Core Model. The types of information were discussed and categorized by the authors jointly after reviewing the Core Model. The level of agreement between the identified types of information and the topics included under each domain in the Core Model was taken into consideration when categorizing the information.

The different types of information were seldom clearly defined in the literature, making it difficult to assess whether informational needs with very similar wording had the same meaning. Therefore, even when the identified types of information appeared to be very similar, the types were not merged unless their wording was exactly the same. The lack of clear definitions also meant that some types of information could be interpreted in different ways and could be categorized under two different domains.

### Ranking of Importance of Information Types

The relative importance of the identified types of information was determined according to (i) the number of different information types within each domain, and (ii) the frequency with which the information types within each domain were mentioned in the literature. This second approach based on the frequency of mentions in the literature has been used in previous studies (6;7). The results were compared with the results of studies in which hospital decision makers were explicitly asked to assess the relative importance of different types of information.

## RESULTS

### Identification of Information Types

**Figure 1.**
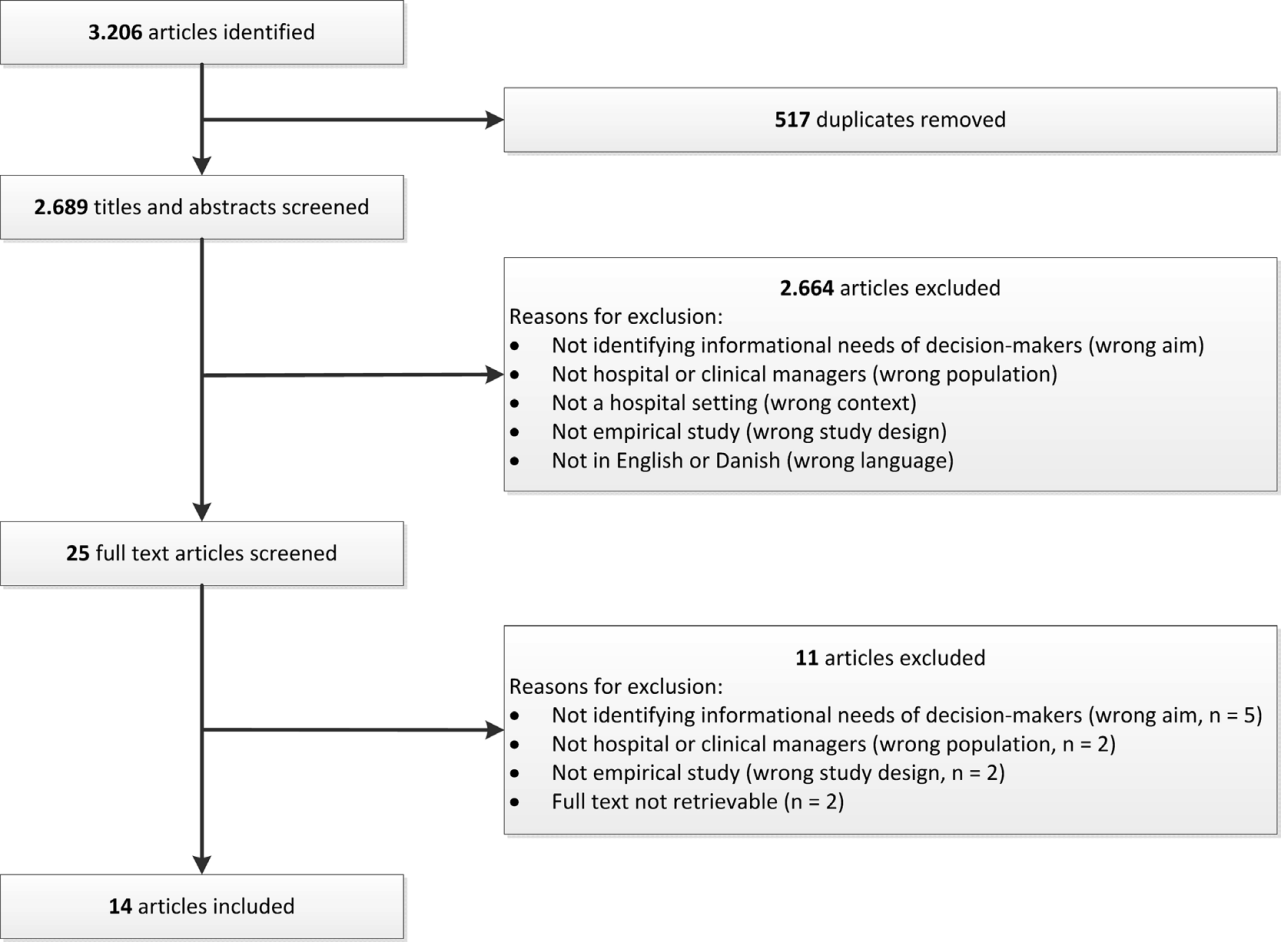
Flow-chart of the systematic literature review, including reasons for exclusion of articles.

The combined search strategies identified 2,689 articles, from which 2,664 were excluded after review of the title and abstract. The remaining twenty-five full-text articles were reviewed and fourteen empirical studies or reviews of empirical studies were considered relevant and included in the analysis (Figure 1). Relevant characteristics of the included literature are presented in Table 1.

**Table 1.** Characteristics of the Studies Included in the Systematic Review and Their Findings Regarding Decision-Makers’ Need for Information.

Study	Country	Objectives	Population (n)	Materials and methods	Results
1. Gagnon MP et al. (8).	Canada	To explore the practices, perceptions and views of HTA stakeholders concerning patient involvement in HTA at the local level.	1) 24 HTA producers and managers, and 2) 13 patient representatives.	Semi-structured interviews and focus group interviews.	Patient involvement is considered relevant and important. The more impact on patient life, the more involvement. When decision making/context/technology requires looking at implementation issues, acceptability, organization of services, training, knowledge about adoption of recommendations are important. NOT only looking at costs and efficiency
2. Parker LE et al. (9).	USA	To explore the views regarding what constitutes evidence and relative importance of evidence vs. practical needs when determining clinical policy	Executive-level Veteran Health Administration policymakers. n = 26.	Semi-structured interviews and content analysis.	What constitutes the relative importance of evidence versus practical needs when determining clinical policy: 1) Practical in real world clinical setting, 2) Fit with values and local circumstances and context, 3) Resources, 4) Respond to political climate, 5) Meet compelling patient needs (volume, high-risk), 6) Fidelity (doing things just like in the trial implemented).
3. Ratcliffe J et al. (10).	UK	To describe the views of NHS decision makers concerning the concept of cost-effectiveness, equity and access, and to examine trade-offs between the importance of the concepts.	Health care decision makers in the NHS. n = 380.	Attitudinal questionnaires with 25 statements on a (1–6) Likert scale.	Many respondents were prepared to trade between attributes related to cost-effectiveness, equity and access. Clinical decision-makers are more concerned about access and less about equity than decision makers at higher levels. 75% agree that cost-effectiveness is essential when allocating resources.
4. Hivon M et al. (11).	Canada	To define more precisely how HTA is used by interviewees as well as the most significant barriers they encounter.	1) Health care administrator associations and governments, 2) Health care provider associations, 3) Patient associations. n = 42	Semi-structured interviews, qualitative analysis	The vast majority recognize the usefulness of HTA. HTA use takes many forms: 1) Instrumental: implementation of services and programs, 2) Conceptual: framework to stimulate debate and orient government policies and 3) Symbolic: enforcing decisions already made. Significant barriers hinder the use of HTA: 1) Organizational: pure in-house communications, lack of long term planning, decision makers vested interests, 2) Scientific: absence of skilled staff and 3) Material: lack of time, human + material + financial resources.
5. Sampietro-Colom L et al. (2).	Spain	To develop and test a decision-support tool for prioritizing new competing HTs after their assessment using the mini-HTA approach.	28 decision-makers at national/regional level and hospital level.	A two-layer value/risk tool was developed based on the mini-HTA. The first layer included 12 mini-HTA variables classified in two dimensions—value and risk. Weights given to these were obtained from a questionnaire survey among decision-makers (9 point Likert scale).	12 mini-HTA variables: *Values* (clinical and patient implications): Safety, clinical benefits, patient impact, cost-effectiveness, quality of evidence, innovativeness. *Risk* (impact on management dimension): Staff requirements, physical space impact, process of care impact, incremental cost, net cost, investment effort. No significant differences among decision-makers at micro (hospital) and macro level were observed as regards the weights given to the 12 variables. Importance/weights (in parentheses: authors’ assessment of relevant EUnetHTA domain): 1. Quality of evidence (D3) 2. Safety (D4), clinical benefit (D3), cost-effectiveness (D5) 3. Patient impact (D3), innovativeness (D2), process of care impact (D7)
6. Gibson JL et al. (12).	Canada	To facilitate work-shops for board members and senior leadership at three health care organizations to assist them in developing a strategy for fair priority setting.	Board members and senior administrators/leadership at three health care organizations.	One-day workshop with case-based plenary sessions.	8 priority setting criteria: 1) Strategic fit, 2) Alignment with external directives, 3) Academic commitments, 4) Clinical impact, 5) Community need, 6) Partnerships, 7) Interdependency, 8) Resource implications. Efficiency considerations or simple technical solutions have only limited influence on decision-making and are not sufficient alone to guide decision-making.
7. Niedzwiedzka BM (13).	Poland	To obtain data describing the needs, preferences and limitations of healthcare managers as information users, and to identify environmental factors influencing their information behavior.	Hospital chief executives, medical directors, head nurses and directors of self-government health departments. n = 815.	Questionnaire survey, focus groups, semistructured interviews, analysis of policy documents.	Cost-effectiveness analysis of interventions, clinical practice guidelines and data about local health priorities were consistently declared relevant by all subgroups. There are differences in the hierarchy of needs in subgroups. National strategies are considered most needed by most CEO. Local priorities are considered least needed.
8. Greenberg D et al. (14).	Israel	To describe the considerations relevant to decision makers when making decision about acquisition of new technology at the hospital level.	Hospital directors, vice directors, administrative directors. n = 29.	Literature review, in-depth interviews, questionnaire (6 point Likert scale + ranking: rank-order top 5 most/less important criteria).	*Importance/Likert scale* (in parentheses: authors’ assessment of relevant EUnetHTA domain): 1. Clinical considerations (D3) 2. Impact on hospitals profitability and other economic components (D5) 3. Need for personnel training (D7) 4. Available information regarding the new technology (D2) 5. Formal approvals (D9) *Ranking:* *Top relevant criteria:* need for a large capital investment, clinical efficacy of the technology, influence on side effects and complication rates, formal approval of MOH. *Less relevant criteria:* demands and pressures exerted by industry, patients, senior physicians.
9. Gallego G et al. (15).	Australia	To investigate the perceptions, concerns and attitudes of decision-makers regarding access to high cost medicines (HCMs) in public hospitals.	Executive directors of hospitals, area health service managers, directors of hospital pharmacy departments and senior medical doctors. n = 24.	In-depth semi-structured interviews.	Decisions are based on criteria such as safety, efficacy, effectiveness, costs and budgetary constraints, quality of life, clinical needs, lack of alternative treatment, cost-effectiveness. In general, respondents referred solely to costs and budgetary constraints rather than costs and benefits together. Cost-effectiveness is a ‘desired’ criterion but not really considered in practice.
10. Andradas E et al. (16).	Spain	To explore the needs and requirements of decision makers in our regional healthcare system for health technology assessment (HTA) products to support portfolio development for a new HTA agency in Madrid, Spain.	87 managers, medical directors and general directors from 21 public hospitals, 11 primary healthcare centers, 6 private hospitals, and 8 departments of the Regional MoH. Response rate = 83.9 % (n = 73/87).	Delphi study with two rounds. Semi-structured questions.	Public hospitals and primary healthcare centers preferred classic technology-centered HTA products such as HTA reports, systematic reviews, economic assessments and drug and medical/surgical procedure assessments, rapid reviews (micro perspective), whereas private hospitals and ministry representatives demanded, in addition to the requirement for classic and more innovative HTA products, more innovative HTA products such as organizational model and information system assessments, HTA research projects (macro perspective).
11. Vuorenkoski L et al. (17).	Finland	To review studies that empirically analyze macro and meso level decision making process for Including and/or excluding drugs in reimbursement lists and drug formularies in industrialized countries.		Review of 6 qualitative empirical studies	Clinical evidence on benefit and the quality of evidence were the main criteria. The costs of the drug emerged as the second major criteria. Formal pharmacoeconomic analyses had a minor role. Other criteria used by decision-makers were alternative treatments available, decision in other hospitals/systems, size of population affected, severity of disease and past decisions. External factors mentioned as influencing decision-making were patient demand, pharmaceutical company activities and clinicians’ excitement. The criteria used varied between studies, and also between decisions.
12. Galani C et al. (6).	Netherlands	1) To summarize published literature on self-reported attitudes of health care decision makers towards economic evaluations of medical technologies, and 2) To examine the extent to which economic evaluations are used in health policy decisions.		Systematic literature review of 55 qualitative empirical studies investigating the attitudes toward economic evaluations among decision-makers and actual use patterns.	Participants found economic evaluations useful to inform policy decisions. The impact on policy s reported moderate in the majority of studies. Clinical aspects such as efficacy and effectiveness, and safety data were still considered the most influential arguments. Different aspects characterized each decision level: central = regulatory and political arguments, local = economics, physician = patient, disease and administrative burden.
13. Ehlers L et al. (18).	Denmark	To evaluate local decision support tools used in the Danish hospital sector from a theoretical and an empirical point of view.	1) County health directors, 2) Hospital managers and 3) Heads of clinical departments in cardiology, orthopedic surgery and anesthesiology. n = 139.	3 different mailed questionnaires to the 3 groups of respondents. A theoretical analysis of decision support tools was performed. Danish mini-HTA was compared with foreign production and use of HTA and HTA-like assessments. International experience with the mini-HTA was reviewed in a literature study + 11 local/regional HTA organizations and 14 individuals were contacted.	Mini-HTA is being used by 55 % of the hospital authorities, 66 % of the hospital management sections and 27 % of the department management teams. *Advantages of mini-HTA*: rest on evidence-based knowledge, interdisciplinary overall assessments oriented toward decision-making problems, standardization, timing. *Disadvantages of mini-HTA*: insufficiency of evidence-base, lack of quality control, administrative burden. No decision makers based their decisions exclusively on mini-HTAs, only a supplement.
14. Greenberg D et al (3).	Israel	1) To map and describe the function of hospital decision-makers within the area of new technology assessment and adoption, 2) to examine relevant considerations, sources of information and decision-making processes in the adoption of new technology.	132 hospital executives.	Mailed questionnaire (ranking: rank-order top 5 criteria for/against adoption) based on a comprehensive literature review and in-depth interviews with decision-makers.	*Top criteria for adoption:* increased cost-effectiveness, increased efficacy, decreased complication rates. *Top criteria against adoption:* increased complication rates or side effects, decreased efficacy. It is optimal to use specialized medical journals, opinions of international leaders in a specific field, evidence from RCTs, large prospective studies and meta-analyses. *Importance/frequency of criteria for/against adoption* (in parentheses: authors’ assessment of relevant EUnetHTA domains: 1. Cost-effectiveness (D5) 2. Efficacy (D3) 3. Complication rates/side-effects (D4) 4. Ministry of health approval (D9/D10) 5. Technology is still experimental (D1/D2)

Reasons for exclusion were that (i) the aim of the study was not to identify informational needs of decision makers (wrong aim), (ii) the study did not deal with decision making by hospital or clinical managers (wrong population), (iii) the study did not take place in a hospital setting (wrong context), (iv) the article was not based on an empirical study or was based on an empirical study of low quality (wrong/poor study design), (v) the article was not in English or Danish (wrong language), or (vi) the article was not retrievable (*n* = 2). The kappa coefficient was 0.54, indicating moderate agreement between reviewers in the initial assessment of articles.

Half of the fourteen included studies were conducted in Canada, Spain, and Israel, and the rest were conducted in other European countries, United States, and Australia. The study methodologies varied, but typically included semi-structured interviews, questionnaire surveys and literature reviews. From the fourteen articles, we identified seventy-four different types of information requested by hospital and clinical managers.

### Categorization of Informational Types

Most of the seventy-four types of information could be categorized within the nine domains of EUnetHTA's Core Model (Table 2). Due to ambiguity in wording, fifteen (20.3 percent) of the seventy-four information types were categorized under a maximum of two domains.

**Table 2.** Categorization of information Types According to Ten Domains, and the Frequency with Which These Types Were Mentioned in the Fourteen Articles Included in the Systematic Review

Domain	No. of information types in domain	Types of information from literature (frequency of each information type)		Frequency sum for all information types in domain
D1: Health problem and current use of the technology*	10	· Community need (1) · Meet compelling patient need (volume, high-risk), (1) · Clinical needs (1) · (Lack of) alternative treatment (2) · Size of population affected/patient volume (2)	· Severity of disease (1) · Technology is still experimental (maturity), (1) · Waiting time (1) · Past decisions (1) · Demand and pressures exerted by patients (2)	13
D2: Description and technical characteristics of technology*	6	· Innovativeness (1) · Available information regarding the new technology (1) · Feasibility (1)	· Technology is still experimental (maturity), (1) · Technology could be applied on an outpatient basis (1) · Physical space impact (1)	6
D3: Clinical effectiveness*	12	· Clinical benefit (2) · Patient impact (1) · Quality of evidence (2) · Clinical impact (1) · Real world clinical setting (2) · Clinical considerations and implications (1) · Efficacy of new technology compared to existing technology (4)	· Sufficient data in medical literature regarding the results of clinical trials (2) · Effectiveness (2) · Quality of life (2) · Waiting time (1) · Fidelity (to the trial), (1)	21
D4: Safety aspects*	4	· Safety (3) · Side effects by the use of technology (2)	· Changes in complication rates (2) · Invasiveness of technology (1)	8
D5: Costs and economic evaluation*	11	· Cost-effectiveness (5) · Incremental cost (1) · Net cost (1) · Investment effort (1) · Impact on hospital's profitability and other economic components (2) · Technology is associated with large capital investment (2)	· Costs and budgetary constraints (3) · Change in hospital income (1) · Provisions of “out-of-pocket services” (1) · Resource implications (1) · Influence on hospital's exploitation (1)	19
D6: Ethical aspects*	3	· Ethical issues (2) · Equity (1)	· Provisions of “out-of-pocket services” (1)	4
D7: Organizational aspects*	15	· Staff requirements (1) · Process of care impact (1) · Partnerships (1) · Interdependency (1) · Implementation issues (1) · Organization of services (1) · Training (1) · Organizational resources (1) · The need for personnel training (2)	· Technology could be applied on an outpatient basis (1) · Physical space impact (1) · Fidelity (to the trial), (1) · Resource implications (1) · Influence on hospital's exploitation (1) · Fit with values and local circumstances and context (1)	16
D8: Social aspects*	5	· Patient's perspective is relevant and important (1) · Acceptability (1) · Distance travelled (1)	· Knowledge about adoption and recommendations (1) · Invasiveness of technology (1)	5
D9: Legal aspects*	7	· Clinical practice guidelines (1) · Formal approvals (1) · Food and Drug Administration approval (1) · Change in liability risk (1)	· Alignment with external directives (1) · Technology approved by the ministry of health (2) · National health strategy and directives (1)	8
D10: Political and strategic aspects	16	· Strategic fit (1) · Academic commitments (1) · Respond to political climate (1) · Local health priorities (1) · Prestige and competition among hospitals (2) · Pressures exerted inside and outside the hospitals (1) · Commercial pressures exerted by the industry (2) · Decisions in other hospitals (cover and use the technology), (2) · Pressures exerted by directors of medical wards or senior physicians (2)	· Clinicians excitement (1) · Demand and pressures exerted by patients (2) · Past decisions (1) · Fit with values and local circumstances and context (1) · National health strategy and directives (1) · Alignment with external directives (1) · Technology approved by the ministry of health (2)	22

*Note.* For example, ten of the information types that were identified in the literature (Table 1) were categorized within Domain 1 (number), and they were mentioned a total of thirteen times (frequency). The shadowed cell indicates a new domain that is not covered by EUnetHTA's Core Model.

Ten (13.5 percent) of the seventy-four information types did not fit easily into any of the domains of the Core Model and were categorized under a new, tenth domain entitled “political and strategic aspects” (Table 2). Six of the fifteen (40.0 percent) information types that were not clearly defined were categorized under both the new, tenth domain and one of the other domains. Thus, a total of sixteen (21.6 percent) information types were categorized under the new domain covering “political and strategic aspects” of HT investments (Table 2). Political aspects referred to, for example, the alignment between the decision to invest in a given technology and the local political climate or values. Strategic aspects referred to, for example, the fit between a given technology and the hospital's research strategy, as well as prestige and competition between hospitals in relation to a specific technology or health problem.

The third domain (D3) dealing with “clinical effectiveness,” included information about clinical outcomes (e.g., quality of life) and effect sizes (e.g., patient impact) as well as characteristics of the evidence (e.g., quality of evidence).

The fifth domain (D5), dealing with “costs and economic evaluation,” included information from traditional health economic evaluations with a broad societal perspective (e.g., cost-effectiveness analysis) as well as narrower budget impact analyses with a hospital perspective (e.g., business cases).

The domains dealing with ethical (D6), safety (D4), and social (D8) aspects had the fewest number of information types (Table 2). Information on the patient perspective appeared only once in the literature (Domain 8 in Table 2).

### Ranking of Importance of Information Types

Regardless of the approach used to rank the domains in terms of importance (i.e. number of different information types vs. frequency of mentions in the literature), the same five domains were ranked as the most important, albeit in differing order (Table 2). These domains were those dealing with information about political and strategic aspects (D10), clinical aspects (D3), economic aspects (D5), organizational aspects (D7), and the health problem and current use of the technology (D1). Mainly information about organizational aspects changes from being the second most important type of information when looking at the number of different information types within each domain (column 2 in Table 2) to being the fourth most important type of information when looking at the frequency of mentions in the literature (column 4 in Table 2).

Three of the fourteen reviewed articles described studies in which hospital decision makers were directly asked to rank different types of information in order of their relative importance—one study from Spain (2) and two from Israel (3;14). These studies used Likert scales, rank-order, or both to assign importance.

Most of the information types that emerged from these studies as being important came under the same five domains that we identified as being important based on frequency of mentions in the literature. However, information about political and strategic aspects (D10) was rarely mentioned as important in the three direct measurement studies. Furthermore, a description of the technology and its technical characteristics (D2) and information about safety (D4) and legal aspects (D9) were ranked as important aspects in the direct measurement studies, but were not identified as important using our approaches. Of note, the social (D8) and ethical (D6) aspects of a new technology were rarely considered as important information for decision making.

## DISCUSSION

This systematic review identified fourteen relevant empirical studies that contained seventy-four different types of information requested by hospital decision makers when deciding on HT investments. The EUnetHTA guidelines for performing HTA, that is, the nine domains of the Core Model (4), included most of these different types of information (4). However, we identified types of information that related to a new, tenth domain covering “political and strategic aspects.”

The additional domain dealing with the strategic and political aspects of investments in new health technologies is in line with McGregor's (19) assertion that investment decisions are dependent on political and social pressures and the opinion and values of the hospital decision makers. According to Gray (20) healthcare decisions are based on a combination of three factors: *evidence*, *values*, and *resources*. At present, some decisions may be driven principally by *values* and *resources*—a process Gray (20) describes as *opinion-based decision making*. The new domain dealing with “political and strategic aspects” (D10) cover information that by definition is something else than *evidence*. These types of information are, however, requested by hospital decision makers and, therefore, it may be worth considering including information on these more value-based aspects of HT investments as part of a basis for decision making in hospitals.

We found that information about political and strategic aspects of new technology (D10), together with clinical (D3), economic (D5), and organizational (D7) aspects were mentioned most frequently in the literature and were also those domains with the highest number of different information types. The importance of information about clinical effectiveness and economic aspects was confirmed in the three direct measurement studies, but not information about political and strategic aspects (D10). The reason may be that the hospital decision makers were not directly asked to consider these (new) aspects of HT investments in these studies. A recent systematic review of decision criteria for resource allocation and healthcare decision making showed that the most frequently cited category of criteria was “Overall context” including (among others) political aspects and stakeholders interests and pressures (7). It does seem then, that consideration of the political and strategic aspects are important in hospital decision making.

Our results suggested that hospital decision makers rarely focus on information about ethical (D6), safety (D4), social (D8), and legal (D9) aspects of new HT. This may be due to less familiarity with this type of information or with the way in which the information (especially about legal and ethical aspects) is collected. It may be that safety information was assumed to be included in the domain on clinical outcomes and effectiveness (D3). Furthermore, it was somewhat surprising that information on patient satisfaction and patient preferences was rarely directly requested, given that the patient perspective is one of four main categories in the Danish template for mini-HTA (21).

Future studies on the relative importance of different types of information to hospital decision makers should distinguish between information on clinical outcomes and effect sizes on the one hand, and information on the characteristics and quality of the evidence on the other. Both of these types of information were categorized here within the domain of clinical effectiveness (D3), but it is possible that hospital decision makers weight these types of information differently. Quality of evidence is a key determinant for the strength of recommendations for or against the adoption of a given HT (22) and this type of information was considered the most important in one of the three direct measurement studies (2).

It may also be relevant to examine more closely the relative importance of the broad societal perspective and the narrower hospital perspective in economic analyses of HT. Most of the information types identified from the literature related to the local hospital perspective, but investigating the relative importance of different perspectives when directly asking hospital decision makers to prioritize between information in future studies will be interesting. McGregor (19) noted that cost-effectiveness of a given technology does not determine investment decisions, but budget impact does. Similarly, Gallego et al. (15) found that information on the local budget impact of a new technology was more often requested by hospital decision makers than conventional economic evaluations with a broad societal perspective.

Likert scales and rank ordering exercises were used in the three direct measurement studies (Table 1). The Spanish study used only a Likert scale, and the results showed that the decision makers mostly agreed that all the types of information were important to some extent (2). The use of Likert scales alone does not force decision makers to prioritize between different types of information. A combination of Likert scales and ranking (14) is, therefore, recommended in future studies.

A recent international questionnaire survey invited healthcare decision makers to report which criteria they consider when making decisions on healthcare interventions (22). Respondents were asked to indicate whether each decision criterion was “currently considered” or “should be considered” and its relative weights. The most relevant criteria were found to be: (i) clinical efficacy/effectiveness, (ii) safety, (iii) quality of evidence, (iv) disease severity, and (v) impact on healthcare costs. Organizational and skill requirements were frequently considered, but had relatively low weights, suggesting that their impact on the final decision might be fairly small. These results are largely consistent with the results of the three direct measurement studies in the current review.

We found that EUnetHTA's Core Model did not include all the types of information considered in hospital decision making. What are the implications of this for HB-HTA? Should guidelines for HB-HTA be adjusted and thereby differ from those for full HTA, for example, EUnetHTA's Core Model?

One possibility is that HB-HTA should focus exclusively on the clinical, economic, safety, organizational, strategic, and political issues associated with the introduction and use of a specific HT. McGregor (19) suggests that there is often a disconnection between the rigorous and careful collection of evidence as part of the HTA process, and the failure of this process to influence policy decisions. This is partly due to the HTA often being delivered too late for inclusion in the final basis for decisions. Results from a Polish study suggested that healthcare managers favor speed over accuracy of information in evidence-based decision making (13). A more focused and targeted approach to assessing HT, leaving out, for example, ethical and social aspects not valued highly by hospital decision makers, might allow a faster and less resource-consuming assessment at hospital level.

Further research is needed before concluding anything definitive about the practical implications for HB-HTA. We need more knowledge about what hospital decision makers understand by strategic and political aspects and what importance they place on the quality of clinical evidence and the different perspectives used in economic analyses of HT. These issues will be among those investigated in the interview study and the questionnaire survey among hospital decision makers in Europe that are part of the next steps in the AdHopHTA project.

### Methodological Considerations

Several methodological considerations need to be taken into account when interpreting our results. First, the literature search was restricted to articles in English and Danish, and relevant literature in other languages could have been missed. In fact, no relevant literature in Danish was identified, so this language choice had no impact on the final results. In addition, we could not retrieve two potentially relevant articles.

Second, although all the reviewed studies included hospital managers and/or clinical managers, it was not always possible to isolate their results from those of other decision makers included in the study. Some studies were conducted at hospital level (micro), others at regional (meso) and national (macro) levels of decision making. Thus, we cannot be sure that our results are based solely on the informational needs of hospital and clinical managers.

Third, the included articles had very different purposes and research questions. Some articles investigated the relevance of a single criterion or element (e.g., the patients’ perspectives) or particular product (e.g., mini-HTA or economic evaluation), while others focused on decision making in relation to a specific type of technology (e.g., expensive pharmaceuticals).The included literature also involved different study methodologies (typically systematic reviews, semi-structured interviews, and questionnaire surveys) and very different sample sizes, which is consistent with the methods used in the AdHopHTA project. This might, however, have influenced the importance ranking of the ten domains based on the number of information types or the frequency of their mentions in the literature, and these results should, therefore, be interpreted with caution. Notice, that the three direct measurement studies were among the fourteen relevant articles included in this review (Study no. 5, 8, and 14 in Table 1).

Fourth, the literature used a variety of concepts to describe the information that decision makers need when deciding whether or not to invest in HT, including, for example, “decision support,” “information,” and “decision criteria.” We have not distinguished between these concepts, and this study thus concerns the need for “information” among hospital decision makers, which is a wider concept than the specific and measurable “criteria”.

Fifth, the different types of information were discussed and categorized jointly by the authors after a thorough review of the Core Model. It would have been preferable for the authors to categorize the information independently before having a joint discussion. However, the result of the categorization was subsequently discussed with and validated by a group of HTA experts in the AdHopHTA project, which enhances the quality of the analysis (please see http://www.adhophta.eu/ for further details about the AdHopHTA project).

Because the different types of information were seldom clearly defined in the literature, we found fifteen of them to be sufficiently ambiguous that they had to be categorized under two domains. For example, information on the level of maturity of the technology (“Technology is still experimental”) could be placed either in domain 1 (D1: Health problem and current use of the technology”) or domain 2 (D2: Description and technical characteristics of the technology). Similarly, information about the effect of a specific technology on the exploitation of the hospital (“Influence on hospital's exploitation”) could be categorized under domain 5 (D5: Costs and economic evaluation) or domain 7 (D7: Organizational aspects). Even when we forced each of these fifteen ambiguous types of information into one domain only, the results remained largely unchanged. Thus, the most important types of information were still within the same five domains. The only difference was that information on organizational aspects (D5) went from being the third most important to the second most important information together with information on clinical aspects (D3) when using the number of information types as a measure of the relative importance.

Finally, the included literature was based on research conducted in ten different countries. Results cannot be transferred uncritically from one context to another because of national differences in healthcare systems, decision-making processes, and attitudes toward the use of HTA.

## CONCLUSION

The results of this systematic review suggest that hospital decision makers are able to describe their informational needs when deciding on HT investments. The domains of EUnetHTA's Core Model appeared to cover most of the informational needs of hospital and clinical managers, but full agreement was not observed. In addition to the domains of the Core Model, hospital decision makers also seek information on strategic and political aspects not covered by the model. Furthermore, the domains are not of equal importance to hospital decision makers. Clinical, economic, and strategic/political aspects are mentioned most frequently in the literature. The importance of clinical and economic aspects is confirmed in studies of relative importance of different types of information among hospital decision makers. Finally, this literature review also shows that the relative importance that hospital decision makers assign to different types of information has seldom been examined.

The results of this systematic review provide further knowledge about the types of information that hospital decision makers consider relevant when they decide on HT investments. This information will be useful for directing future empirical studies on this subject, including the interview study and questionnaire survey that will be conducted in the next phase of the AdHopHTA project.

## Supplementary material

For supplementary material accompanying this paper visit http://dx.doi.org/10.1017/S0266462315000665. click here to view supplementary material

## CONFLICTS OF INTEREST

Dr. Ølholm reports grants from EC Seventh Framework Programme, during the conduct of the study. Drs. Kidholm, Birk-Olsen, and Buck Christensen have nothing to disclose.
