# Supplementary material for: HOSPITAL MANAGERS’ NEED FOR INFORMATION ON HEALTH TECHNOLOGY INVESTMENTS
Source: Int J Technol Assess Health Care. 2015;31(6):414–25. doi: 10.1017/S0266462315000665 (PMC4824957; doi:10.1017/S0266462315000665)
Supplement: Supplementary file 1 [file S0266462315000665sup001.docx]

***SUPPLEMENTARY MATERIAL***

Supplementary Table 1: Full search histories

PubMed (15.11.12)

| **Search** | **Query** | **Items found** |
| --- | --- | --- |
| [#97](http://www.ncbi.nlm.nih.gov/pubmed/advanced) | Search (#94 AND #95) Filters: Publication date from 2000/01/01 to 2012/12/31 | [1274](http://www.ncbi.nlm.nih.gov/pubmed/?cmd=HistorySearch&querykey=97) |
| [#96](http://www.ncbi.nlm.nih.gov/pubmed/advanced) | Search (#94 AND #95) | [1948](http://www.ncbi.nlm.nih.gov/pubmed/?cmd=HistorySearch&querykey=96) |
| [#95](http://www.ncbi.nlm.nih.gov/pubmed/advanced) | Search (#47 AND #68 AND #73) | [3573](http://www.ncbi.nlm.nih.gov/pubmed/?cmd=HistorySearch&querykey=95) |
| [#94](http://www.ncbi.nlm.nih.gov/pubmed/advanced) | Search (#75 OR #77 OR #79 OR #81 OR #83 OR #84 OR #85 OR #86 OR #87 OR #88 OR #89 OR #90 OR #91 OR #92 OR #93) | [3685790](http://www.ncbi.nlm.nih.gov/pubmed/?cmd=HistorySearch&querykey=94) |
| [#93](http://www.ncbi.nlm.nih.gov/pubmed/advanced) | Search ("stated preference") OR "stated preferences" | [241](http://www.ncbi.nlm.nih.gov/pubmed/?cmd=HistorySearch&querykey=93) |
| [#92](http://www.ncbi.nlm.nih.gov/pubmed/advanced) | Search ("revealed preference") OR "revealed preferences" | [67](http://www.ncbi.nlm.nih.gov/pubmed/?cmd=HistorySearch&querykey=92) |
| [#91](http://www.ncbi.nlm.nih.gov/pubmed/advanced) | Search (metaanalysis) OR metaanalyses | [29948](http://www.ncbi.nlm.nih.gov/pubmed/?cmd=HistorySearch&querykey=91) |
| [#90](http://www.ncbi.nlm.nih.gov/pubmed/advanced) | Search (meta analysis) OR meta analyses | [64603](http://www.ncbi.nlm.nih.gov/pubmed/?cmd=HistorySearch&querykey=90) |
| [#89](http://www.ncbi.nlm.nih.gov/pubmed/advanced) | Search (focus group) OR focus groups | [50468](http://www.ncbi.nlm.nih.gov/pubmed/?cmd=HistorySearch&querykey=89) |
| [#88](http://www.ncbi.nlm.nih.gov/pubmed/advanced) | Search (review) OR reviews | [2126645](http://www.ncbi.nlm.nih.gov/pubmed/?cmd=HistorySearch&querykey=88) |
| [#87](http://www.ncbi.nlm.nih.gov/pubmed/advanced) | Search (questionnaire) OR questionnaires | [404528](http://www.ncbi.nlm.nih.gov/pubmed/?cmd=HistorySearch&querykey=87) |
| [#86](http://www.ncbi.nlm.nih.gov/pubmed/advanced) | Search (interview) OR interviews | [192942](http://www.ncbi.nlm.nih.gov/pubmed/?cmd=HistorySearch&querykey=86) |
| [#85](http://www.ncbi.nlm.nih.gov/pubmed/advanced) | Search (survey) OR surveys | [1526956](http://www.ncbi.nlm.nih.gov/pubmed/?cmd=HistorySearch&querykey=85) |
| [#84](http://www.ncbi.nlm.nih.gov/pubmed/advanced) | Search (empirical study) OR empirical studies | [43752](http://www.ncbi.nlm.nih.gov/pubmed/?cmd=HistorySearch&querykey=84) |
| [#83](http://www.ncbi.nlm.nih.gov/pubmed/advanced) | Search "Focus Groups"[Mesh] | [13872](http://www.ncbi.nlm.nih.gov/pubmed/?cmd=HistorySearch&querykey=83) |
| [#81](http://www.ncbi.nlm.nih.gov/pubmed/advanced) | Search "Meta-Analysis" [Publication Type] | [35488](http://www.ncbi.nlm.nih.gov/pubmed/?cmd=HistorySearch&querykey=81) |
| [#79](http://www.ncbi.nlm.nih.gov/pubmed/advanced) | Search "Review" [Publication Type] | [1721247](http://www.ncbi.nlm.nih.gov/pubmed/?cmd=HistorySearch&querykey=79) |
| [#77](http://www.ncbi.nlm.nih.gov/pubmed/advanced) | Search "Questionnaires"[Mesh] | [266275](http://www.ncbi.nlm.nih.gov/pubmed/?cmd=HistorySearch&querykey=77) |
| [#75](http://www.ncbi.nlm.nih.gov/pubmed/advanced) | Search "Interview" [Publication Type] | [23020](http://www.ncbi.nlm.nih.gov/pubmed/?cmd=HistorySearch&querykey=75) |
| [#73](http://www.ncbi.nlm.nih.gov/pubmed/advanced) | Search (#70 OR #71 OR #72) | [2600266](http://www.ncbi.nlm.nih.gov/pubmed/?cmd=HistorySearch&querykey=73) |
| [#72](http://www.ncbi.nlm.nih.gov/pubmed/advanced) | Search (hospital setting) OR hospital settings | [139629](http://www.ncbi.nlm.nih.gov/pubmed/?cmd=HistorySearch&querykey=72) |
| [#71](http://www.ncbi.nlm.nih.gov/pubmed/advanced) | Search (hospital) OR hospitals | [2600266](http://www.ncbi.nlm.nih.gov/pubmed/?cmd=HistorySearch&querykey=71) |
| [#70](http://www.ncbi.nlm.nih.gov/pubmed/advanced) | Search "Hospitals"[Mesh] | [187603](http://www.ncbi.nlm.nih.gov/pubmed/?cmd=HistorySearch&querykey=70) |
| [#68](http://www.ncbi.nlm.nih.gov/pubmed/advanced) | Search (#49 OR #51 OR #52 OR #53 OR #54 OR #57 OR #58 OR #59 OR #60 OR #61 OR #62 OR #63 OR #64 OR #65 OR #66 OR #67) | [489119](http://www.ncbi.nlm.nih.gov/pubmed/?cmd=HistorySearch&querykey=68) |
| [#67](http://www.ncbi.nlm.nih.gov/pubmed/advanced) | Search ("decision making") OR decisionmaking | [160213](http://www.ncbi.nlm.nih.gov/pubmed/?cmd=HistorySearch&querykey=67) |
| [#66](http://www.ncbi.nlm.nih.gov/pubmed/advanced) | Search "priority setting" | [1184](http://www.ncbi.nlm.nih.gov/pubmed/?cmd=HistorySearch&querykey=66) |
| [#65](http://www.ncbi.nlm.nih.gov/pubmed/advanced) | Search (prioritisation) OR prioritisations | [547](http://www.ncbi.nlm.nih.gov/pubmed/?cmd=HistorySearch&querykey=65) |
| [#64](http://www.ncbi.nlm.nih.gov/pubmed/advanced) | Search (prioritization) OR prioritizations | [2683](http://www.ncbi.nlm.nih.gov/pubmed/?cmd=HistorySearch&querykey=64) |
| [#63](http://www.ncbi.nlm.nih.gov/pubmed/advanced) | Search (informational need) OR informational needs | [1061](http://www.ncbi.nlm.nih.gov/pubmed/?cmd=HistorySearch&querykey=63) |
| [#62](http://www.ncbi.nlm.nih.gov/pubmed/advanced) | Search (information need) OR information needs | [77980](http://www.ncbi.nlm.nih.gov/pubmed/?cmd=HistorySearch&querykey=62) |
| [#61](http://www.ncbi.nlm.nih.gov/pubmed/advanced) | Search (decision input) OR decision inputs | [3126](http://www.ncbi.nlm.nih.gov/pubmed/?cmd=HistorySearch&querykey=61) |
| [#60](http://www.ncbi.nlm.nih.gov/pubmed/advanced) | Search (technology adoption) OR technology adoptions | [2924](http://www.ncbi.nlm.nih.gov/pubmed/?cmd=HistorySearch&querykey=60) |
| [#59](http://www.ncbi.nlm.nih.gov/pubmed/advanced) | Search (economic analysis) OR economic analyses | [172784](http://www.ncbi.nlm.nih.gov/pubmed/?cmd=HistorySearch&querykey=59) |
| [#58](http://www.ncbi.nlm.nih.gov/pubmed/advanced) | Search (economic evaluation) OR economic evaluations | [76633](http://www.ncbi.nlm.nih.gov/pubmed/?cmd=HistorySearch&querykey=58) |
| [#57](http://www.ncbi.nlm.nih.gov/pubmed/advanced) | Search ((mini HTA) OR mini HTAs) OR mini HTA's | [16](http://www.ncbi.nlm.nih.gov/pubmed/?cmd=HistorySearch&querykey=57) |
| [#54](http://www.ncbi.nlm.nih.gov/pubmed/advanced) | Search ((HTA) OR HTAs) OR HTA's | [2219](http://www.ncbi.nlm.nih.gov/pubmed/?cmd=HistorySearch&querykey=54) |
| [#53](http://www.ncbi.nlm.nih.gov/pubmed/advanced) | Search (health technology assessment) OR health technology assessments | [14842](http://www.ncbi.nlm.nih.gov/pubmed/?cmd=HistorySearch&querykey=53) |
| [#52](http://www.ncbi.nlm.nih.gov/pubmed/advanced) | Search "Decision Making"[Mesh] | [107150](http://www.ncbi.nlm.nih.gov/pubmed/?cmd=HistorySearch&querykey=52) |
| [#51](http://www.ncbi.nlm.nih.gov/pubmed/advanced) | Search "Technology Assessment, Biomedical"[Mesh] | [8744](http://www.ncbi.nlm.nih.gov/pubmed/?cmd=HistorySearch&querykey=51) |
| [#49](http://www.ncbi.nlm.nih.gov/pubmed/advanced) | Search "Program Evaluation"[Mesh] | [50278](http://www.ncbi.nlm.nih.gov/pubmed/?cmd=HistorySearch&querykey=49) |
| [#47](http://www.ncbi.nlm.nih.gov/pubmed/advanced) | Search (#29 OR #31 OR #33 OR #34 OR #35 OR #36 OR #37 OR #38 OR #39 OR #40 OR #41 OR #42 OR #43 OR #44 OR #45 OR #46) | [30495](http://www.ncbi.nlm.nih.gov/pubmed/?cmd=HistorySearch&querykey=47) |
| [#46](http://www.ncbi.nlm.nih.gov/pubmed/advanced) | Search ("healthcare stakeholder") OR "healthcare stakeholders" | [998](http://www.ncbi.nlm.nih.gov/pubmed/?cmd=HistorySearch&querykey=46) |
| [#45](http://www.ncbi.nlm.nih.gov/pubmed/advanced) | Search ("health care stakeholder") OR "health care stakeholders" | [1332](http://www.ncbi.nlm.nih.gov/pubmed/?cmd=HistorySearch&querykey=45) |
| [#44](http://www.ncbi.nlm.nih.gov/pubmed/advanced) | Search (policymaker) OR policymakers | [4222](http://www.ncbi.nlm.nih.gov/pubmed/?cmd=HistorySearch&querykey=44) |
| [#43](http://www.ncbi.nlm.nih.gov/pubmed/advanced) | Search ("policy maker") OR "policy makers" | [9187](http://www.ncbi.nlm.nih.gov/pubmed/?cmd=HistorySearch&querykey=43) |
| [#42](http://www.ncbi.nlm.nih.gov/pubmed/advanced) | Search (decisionmaker) OR decisionmakers | [190](http://www.ncbi.nlm.nih.gov/pubmed/?cmd=HistorySearch&querykey=42) |
| [#41](http://www.ncbi.nlm.nih.gov/pubmed/advanced) | Search ("decision maker") OR "decision makers" | [6344](http://www.ncbi.nlm.nih.gov/pubmed/?cmd=HistorySearch&querykey=41) |
| [#40](http://www.ncbi.nlm.nih.gov/pubmed/advanced) | Search ("clinical administrative manager") OR "clinical administrative managers" | [322](http://www.ncbi.nlm.nih.gov/pubmed/?cmd=HistorySearch&querykey=40) |
| [#39](http://www.ncbi.nlm.nih.gov/pubmed/advanced) | Search ("clinical manager") OR "clinical managers" | [136](http://www.ncbi.nlm.nih.gov/pubmed/?cmd=HistorySearch&querykey=39) |
| [#38](http://www.ncbi.nlm.nih.gov/pubmed/advanced) | Search ("hospital leader") OR "hospital leaders" | [211](http://www.ncbi.nlm.nih.gov/pubmed/?cmd=HistorySearch&querykey=38) |
| [#37](http://www.ncbi.nlm.nih.gov/pubmed/advanced) | Search ("hospital director") OR "hospital directors" | [168](http://www.ncbi.nlm.nih.gov/pubmed/?cmd=HistorySearch&querykey=37) |
| [#36](http://www.ncbi.nlm.nih.gov/pubmed/advanced) | Search ("hospital manager") OR "hospital managers" | [517](http://www.ncbi.nlm.nih.gov/pubmed/?cmd=HistorySearch&querykey=36) |
| [#35](http://www.ncbi.nlm.nih.gov/pubmed/advanced) | Search ("hospital chief executive officer") OR "hospital chief executive officers" | [1802](http://www.ncbi.nlm.nih.gov/pubmed/?cmd=HistorySearch&querykey=35) |
| [#34](http://www.ncbi.nlm.nih.gov/pubmed/advanced) | Search (("hospital CEO") OR "hospital CEOs") OR "hospital CEO's" | [329](http://www.ncbi.nlm.nih.gov/pubmed/?cmd=HistorySearch&querykey=34) |
| [#33](http://www.ncbi.nlm.nih.gov/pubmed/advanced) | Search ("hospital administrator") OR "hospital administrators" | [6936](http://www.ncbi.nlm.nih.gov/pubmed/?cmd=HistorySearch&querykey=33) |
| [#31](http://www.ncbi.nlm.nih.gov/pubmed/advanced) | Search "Chief Executive Officers, Hospital"[Mesh] | [1773](http://www.ncbi.nlm.nih.gov/pubmed/?cmd=HistorySearch&querykey=31) |
| [#29](http://www.ncbi.nlm.nih.gov/pubmed/advanced) | Search "Hospital Administrators"[Mesh] | [7436](http://www.ncbi.nlm.nih.gov/pubmed/?cmd=HistorySearch&querykey=29) |

Embase (20.11.12)

|  |
| --- |

| **Search** | **Query** | **Items found** | **Search type** |
| --- | --- | --- | --- |
| #1 | exp hospital administrator/ | 8047 | Advanced |
| #2 | hospital administrator.mp. | 8258 | Advanced |
| #3 | hospital administrators.mp. | 1601 | Advanced |
| #4 | hospital administration.mp. | 1735 | Advanced |
| #5 | hospital administrations.mp. | 76 | Advanced |
| #6 | hospital CEO.mp. | 79 | Advanced |
| #7 | hospital CEOs.mp. | 155 | Advanced |
| #8 | hospital CEO's.mp. | 155 | Advanced |
| #9 | hospital chief executive officer.mp. | 18 | Advanced |
| #10 | hospital chief executive officers.mp. | 38 | Advanced |
| #11 | hospital manager.mp. | 65 | Advanced |
| #12 | hospital managers.mp. | 671 | Advanced |
| #13 | hospital director.mp. | 110 | Advanced |
| #14 | hospital directors.mp. | 141 | Advanced |
| #15 | hospital leader.mp. | 8 | Advanced |
| #16 | hospital leaders.mp. | 225 | Advanced |
| #17 | clinical manager.mp. | 57 | Advanced |
| #18 | clinical managers.mp. | 121 | Advanced |
| #19 | clinical administrative manager.mp. | 0 | Advanced |
| #20 | clinical administrative managers.mp. | 0 | Advanced |
| #21 | decision maker.mp. | 1215 | Advanced |
| #22 | decision makers.mp. | 7031 | Advanced |
| #23 | decisionmaker.mp. | 48 | Advanced |
| #24 | decisionmakers.mp. | 221 | Advanced |
| #25 | policy maker.mp. | 166 | Advanced |
| #26 | policy makers.mp. | 10642 | Advanced |
| #27 | policymaker.mp. | 73 | Advanced |
| #28 | policymakers.mp. | 4536 | Advanced |
| #29 | health care stakeholder.mp. | 5 | Advanced |
| #30 | health care stakeholders.mp. | 76 | Advanced |
| #31 | healthcare stakeholder.mp. | 7 | Advanced |
| #32 | healthcare stakeholders.mp. | 46 | Advanced |
| #33 | 1 or 2 or 3 or 4 or 5 or 6 or 7 or 8 or 9 or 10 or 11 or 12 or 13 or 14 or 15 or 16 or 17 or 18 or 19 or 20 or 21 or 22 or 23 or 24 or 25 or 26 or 27 or 28 or 29 or 30 or 31 or 32 | 35548 | Advanced |
| #34 | exp economic evaluation/ | 192131 | Advanced |
| #35 | exp biomedical technology assessment/ | 11410 | Advanced |
| #36 | exp decision making/ | 124050 | Advanced |
| #37 | health technology assessment.mp. | 2140 | Advanced |
| #38 | health technology assessments.mp. | 245 | Advanced |
| #39 | HTA.mp. | 2248 | Advanced |
| #40 | HTAs.mp. | 189 | Advanced |
| #41 | HTA's.mp. | 189 | Advanced |
| #42 | mini HTA.mp. | 12 | Advanced |
| #43 | mini HTAs.mp. | 5 | Advanced |
| #44 | mini HTA's.mp. | 5 | Advanced |
| #45 | economic evaluation.mp. | 11788 | Advanced |
| #46 | economic evaluations.mp. | 2483 | Advanced |
| #47 | economic analysis.mp. | 3968 | Advanced |
| #48 | economic analyses.mp. | 1283 | Advanced |
| #49 | technology adoption.mp. | 244 | Advanced |
| #50 | technology adoptions.mp. | 4 | Advanced |
| #51 | decision input.mp. | 2 | Advanced |
| #52 | decision inputs.mp. | 2 | Advanced |
| #53 | information need.mp. | 230 | Advanced |
| #54 | information needs.mp. | 3036 | Advanced |
| #55 | informational need.mp. | 16 | Advanced |
| #56 | informational needs.mp. | 399 | Advanced |
| #57 | prioritization.mp. | 3238 | Advanced |
| #58 | prioritizations.mp. | 37 | Advanced |
| #59 | prioritisation.mp. | 823 | Advanced |
| #60 | prioritisations.mp. | 4 | Advanced |
| #61 | priority setting.mp. | 1442 | Advanced |
| #62 | decision making.mp. | 229189 | Advanced |
| #63 | decisionmaking.mp. | 1214 | Advanced |
| #64 | 34 or 35 or 36 or 37 or 38 or 39 or 40 or 41 or 42 or 43 or 44 or 45 or 46 or 47 or 48 or 49 or 50 or 51 or 52 or 53 or 54 or 55 or 56 or 57 or 58 or 59 or 60 or 61 or 62 or 63 | 430292 | Advanced |
| #65 | exp hospital/ | 560478 | Advanced |
| #66 | hospital.mp. | 1163129 | Advanced |
| #67 | hospitals.mp. | 180903 | Advanced |
| #68 | hospital setting.mp. | 8094 | Advanced |
| #69 | hospital settings.mp. | 2504 | Advanced |
| #70 | 65 or 66 or 67 or 68 or 69 | 1331980 | Advanced |
| #71 | exp interview/ | 122879 | Advanced |
| #72 | exp questionnaire/ | 341256 | Advanced |
| #73 | exp health care survey/ | 3358 | Advanced |
| #74 | empirical study.mp. | 2427 | Advanced |
| #75 | empirical studies.mp. | 4473 | Advanced |
| #76 | survey.mp. | 935111 | Advanced |
| #77 | surveys.mp. | 78449 | Advanced |
| #78 | interview.mp. | 174024 | Advanced |
| #79 | interviews.mp. | 94695 | Advanced |
| #80 | questionnaire.mp. | 432479 | Advanced |
| #81 | questionnaires.mp. | 104485 | Advanced |
| #82 | review.mp. [mp=title, abstract, subject headings, heading word, drug trade name, original title, device manufacturer, drug manufacturer, device trade name, keyword] | 2536344 | Advanced |
| #83 | reviews.mp. [mp=title, abstract, subject headings, heading word, drug trade name, original title, device manufacturer, drug manufacturer, device trade name, keyword] | 134985 | Advanced |
| #84 | focus group.mp. | 10941 | Advanced |
| #85 | focus groups.mp. | 13249 | Advanced |
| #86 | meta analysis.mp. [mp=title, abstract, subject headings, heading word, drug trade name, original title, device manufacturer, drug manufacturer, device trade name, keyword] | 90710 | Advanced |
| #87 | meta analyses.mp. [mp=title, abstract, subject headings, heading word, drug trade name, original title, device manufacturer, drug manufacturer, device trade name, keyword] | 13630 | Advanced |
| #88 | metaanalysis.mp. [mp=title, abstract, subject headings, heading word, drug trade name, original title, device manufacturer, drug manufacturer, device trade name, keyword] | 2589 | Advanced |
| #89 | metaanalyses.mp. | 668 | Advanced |
| #90 | exp meta analysis/ | 67285 | Advanced |
| #91 | revealed preference.mp. | 54 | Advanced |
| #92 | revealed preferences.mp. | 33 | Advanced |
| #93 | stated preference.mp. | 223 | Advanced |
| #94 | stated preferences.mp. | 119 | Advanced |
| #95 | 71 or 72 or 73 or 74 or 75 or 76 or 77 or 78 or 79 or 80 or 81 or 82 or 83 or 84 or 85 or 86 or 87 or 88 or 89 or 90 or 91 or 92 or 93 or 94 | 3944532 | Advanced |
| #96 | 33 and 64 and 70 | 2378 | Advanced |
| #97 | 95 and 96 | 1155 | Advanced |
| #98 | limit 97 to yr="2000 - 2012" | 821 | Advanced |

Cochrane Library (19.11.12)

| **Search** | **Query** | **Items found** |
| --- | --- | --- |
| #1 | MeSH descriptor: [Hospital Administrators] explode all trees | 9 |
| #2 | MeSH descriptor: [Chief Executive Officers, Hospital] explode all trees | 0 |
| #3 | “hospital administrator” | 5 |
| #4 | “hospital administrators” | 36 |
| #5 | “hospital CEO” | 0 |
| #6 | “hospital CEOs” | 0 |
| #7 | “hospital CEO’s” | 0 |
| #8 | “hospital chief executive officer” | 0 |
| #9 | “hospital chief executive officers” | 0 |
| #10 | “hospital manager” | 0 |
| #11 | “hospital managers” | 10 |
| #12 | “hospital director” | 2 |
| #13 | “hospital directors” | 3 |
| #14 | “hospital leader” | 0 |
| #15 | “hospital leaders” | 1 |
| #16 | “clinical manager” | 1 |
| #17 | “clinical managers” | 3 |
| #18 | “clinical administrative manager” | 0 |
| #19 | “clinical administrative managers” | 0 |
| #20 | “decision maker” | 174 |
| #21 | “decision makers” | 690 |
| #22 | decisionmaker | 4 |
| #23 | decisionmakers | 4 |
| #24 | “policy maker” | 26 |
| #25 | “policy makers” | 908 |
| #26 | Policymaker | 4 |
| #27 | policymakers | 174 |
| #28 | “health care stakeholder” | 0 |
| #29 | “health care stakeholders” | 0 |
| #30 | “healthcare stakeholder” | 0 |
| #31 | “healthcare stakeholders” | 3 |
| #32 | #1 OR #2 OR #3 OR #4 OR #5 OR #6 OR #7 OR #8 OR #9 OR #10 OR #11 OR #12 OR #13 OR #14 OR #15 OR #16 OR #17 OR #18 OR #19 OR #20 OR #21 OR #22 OR #23 OR #24 OR #25 OR #26 OR #27 OR #28 OR #29 OR #30 OR #31 | 1896 |
| #33 | MeSH descriptor: [Program Evaluation] explode all trees | 3945 |
| #34 | MeSH descriptor: [Technology Assessment, Biomedical] explode all trees | 486 |
| #35 | MeSH descriptor: [Decision Making] explode all trees | 2090 |
| #36 | health technology assessment | 21239 |
| #37 | health technology assessments | 21239 |
| #38 | HTA | 11670 |
| #39 | HTAs | 45 |
| #40 | HTA’s | 82 |
| #41 | mini HTA | 76 |
| #42 | mini HTAs | 1 |
| #43 | mini HTA’s | 0 |
| #44 | economic evaluation | 16756 |
| #45 | economic evaluations | 16765 |
| #46 | economic analysis | 17129 |
| #47 | economic analyses | 17223 |
| #48 | technology adoption | 403 |
| #49 | technology adoptions | 403 |
| #50 | decision input | 2583 |
| #51 | decision inputs | 2583 |
| #52 | information need | 17521 |
| #53 | information needs | 17521 |
| #54 | informational need | 128 |
| #55 | informational needs | 128 |
| #56 | prioritization | 145 |
| #57 | prioritizations | 145 |
| #58 | prioritisation | 145 |
| #59 | prioritisations | 145 |
| #60 | “priority setting” | 77 |
| #61 | “decision making” | 4704 |
| #62 | decisionmaking | 21 |
| #63 | #33 OR #34 OR #35 OR #36 OR #37 OR #38 OR #39 OR #40 OR #41 OR #42 OR #43 OR #44 OR #45 OR #46 OR #47 OR #48 OR #49 OR #50 OR #51 OR #52 OR #53 OR #54 OR #55 OR #56 OR #57 OR #58 OR #59 OR #60 OR #61 OR #62 | 51131 |
| #64 | MeSH descriptor: [Hospitals] explode all trees | 2420 |
| #65 | hospital | 130421 |
| #66 | hospitals | 130421 |
| #67 | hospital setting | 27394 |
| #68 | hospital settings | 24480 |
| #69 | #64 OR #65 OR #66 OR #67 OR #68 | 130462 |
| #70 | MeSH descriptor: [Interview] explode all trees | 1 |
| #71 | MeSH descriptor: [Questionnaires] explode all trees | 13886 |
| #72 | MeSH descriptor: [Review] explode all trees | 42 |
| #73 | MeSH descriptor: [Meta-Analysis] explode all trees | 98 |
| #74 | MeSH descriptor: [Focus Groups] explode all trees | 235 |
| #75 | empirical study | 2854 |
| #76 | empirical studies | 2854 |
| #77 | survey | 12731 |
| #78 | surveys | 12731 |
| #79 | interview | 11241 |
| #80 | Interviews | 11241 |
| #81 | questionnaire | 33974 |
| #82 | questionnaires | 33974 |
| #83 | review | 65457 |
| #84 | reviews | 65457 |
| #85 | focus group | 11436 |
| #86 | focus groups | 11436 |
| #87 | meta analysis | 22505 |
| #88 | meta analyses | 22521 |
| #89 | metaanalysis | 317 |
| #90 | metaanalyses | 61 |
| #91 | “revealed preference” | 0 |
| #92 | “revealed preferences” | 1 |
| #93 | “stated preference” | 19 |
| #94 | “stated preferences” | 11 |
| #95 | #70 OR #71 OR #72 OR #73 OR #74 OR #75 OR #76 OR #77 OR #78 OR #79 OR #80 OR #81 OR #82 OR #83 OR #84 OR #85 OR #86 OR #87 OR #88 OR #89 OR #90 OR #91 OR #92 OR #93 OR #94 | 110482 |
| #96 | #32 AND #63 AND #69 | 879 |
| #97 | #95 AND #96 | 847 |
| #98 | #95 AND #96 from 2000 to 2012 | 772 |

NB: We included only the following databases from the Cochrane Library (743 hits):

- Cochrane Reviews: 486 hits
- Other Reviews: 17 hits
- Trials: 5 hits
- Technology Assessments: 8 hits
- Economic evaluations: 227 hits

Web of Science (20.11.12)

| **Search** | **Query** | **Items found** |
| --- | --- | --- |
| # 94 | #93  Databases=SCI-EXPANDED, SSCI, A&HCI, CPCI-S, CPCI-SSH, BKCI-S, BKCI-SSH Timespan=2000-01-01 - 2012-11-21  Lemmatization=On | [360](http://apps.webofknowledge.com.proxy1-bib.sdu.dk:2048/summary.do?product=WOS&doc=1&qid=95&SID=Q2eCMF7If3JniHh6eF2&search_mode=AdvancedSearch) |
| # 93 | #92 AND #91  Databases=SCI-EXPANDED, SSCI, A&HCI, CPCI-S, CPCI-SSH, BKCI-S, BKCI-SSH Timespan=All Years  Lemmatization=On | [409](http://apps.webofknowledge.com.proxy1-bib.sdu.dk:2048/summary.do?product=WOS&doc=1&qid=94&SID=Q2eCMF7If3JniHh6eF2&search_mode=CombineSearches) |
| # 92 | #68 AND #63 AND #30  Databases=SCI-EXPANDED, SSCI, A&HCI, CPCI-S, CPCI-SSH, BKCI-S, BKCI-SSH Timespan=All Years  Lemmatization=On | [764](http://apps.webofknowledge.com.proxy1-bib.sdu.dk:2048/summary.do?product=WOS&doc=1&qid=93&SID=Q2eCMF7If3JniHh6eF2&search_mode=CombineSearches) |
| # 91 | #90 OR #89 OR #88 OR #87 OR #86 OR #85 OR #84 OR #83 OR #82 OR #81 OR #80 OR #79 OR #78 OR #77 OR #76 OR #75 OR #74 OR #73 OR #72 OR #71 OR #70 OR #69  Databases=SCI-EXPANDED, SSCI, A&HCI, CPCI-S, CPCI-SSH, BKCI-S, BKCI-SSH Timespan=All Years  Lemmatization=On | [2416587](http://apps.webofknowledge.com.proxy1-bib.sdu.dk:2048/summary.do?product=WOS&doc=1&qid=92&SID=Q2eCMF7If3JniHh6eF2&search_mode=CombineSearches) |
| # 90 | Topic=("stated preferences")  Databases=SCI-EXPANDED, SSCI, A&HCI, CPCI-S, CPCI-SSH, BKCI-S, BKCI-SSH Timespan=All Years  Lemmatization=On | [405](http://apps.webofknowledge.com.proxy1-bib.sdu.dk:2048/summary.do?product=WOS&doc=1&qid=91&SID=Q2eCMF7If3JniHh6eF2&search_mode=GeneralSearch) |
| # 89 | Topic=("stated preference")  Databases=SCI-EXPANDED, SSCI, A&HCI, CPCI-S, CPCI-SSH, BKCI-S, BKCI-SSH Timespan=All Years  Lemmatization=On | [1273](http://apps.webofknowledge.com.proxy1-bib.sdu.dk:2048/summary.do?product=WOS&doc=1&qid=90&SID=Q2eCMF7If3JniHh6eF2&search_mode=GeneralSearch) |
| # 88 | Topic=("revealed preferences")  Databases=SCI-EXPANDED, SSCI, A&HCI, CPCI-S, CPCI-SSH, BKCI-S, BKCI-SSH Timespan=All Years  Lemmatization=On | [278](http://apps.webofknowledge.com.proxy1-bib.sdu.dk:2048/summary.do?product=WOS&doc=1&qid=88&SID=Q2eCMF7If3JniHh6eF2&search_mode=GeneralSearch) |
| # 87 | Topic=("revealed preference")  Databases=SCI-EXPANDED, SSCI, A&HCI, CPCI-S, CPCI-SSH, BKCI-S, BKCI-SSH Timespan=All Years  Lemmatization=On | [813](http://apps.webofknowledge.com.proxy1-bib.sdu.dk:2048/summary.do?product=WOS&doc=1&qid=87&SID=Q2eCMF7If3JniHh6eF2&search_mode=GeneralSearch) |
| # 86 | Topic=(surveys)  Databases=SCI-EXPANDED, SSCI, A&HCI, CPCI-S, CPCI-SSH, BKCI-S, BKCI-SSH Timespan=All Years  Lemmatization=On | [603004](http://apps.webofknowledge.com.proxy1-bib.sdu.dk:2048/summary.do?product=WOS&doc=1&qid=86&SID=Q2eCMF7If3JniHh6eF2&search_mode=GeneralSearch) |
| # 85 | Topic=(survey)  Databases=SCI-EXPANDED, SSCI, A&HCI, CPCI-S, CPCI-SSH, BKCI-S, BKCI-SSH Timespan=All Years  Lemmatization=On | [603004](http://apps.webofknowledge.com.proxy1-bib.sdu.dk:2048/summary.do?product=WOS&doc=1&qid=85&SID=Q2eCMF7If3JniHh6eF2&search_mode=GeneralSearch) |
| # 84 | Topic=(empirical studies)  Databases=SCI-EXPANDED, SSCI, A&HCI, CPCI-S, CPCI-SSH, BKCI-S, BKCI-SSH Timespan=All Years  Lemmatization=On | [128421](http://apps.webofknowledge.com.proxy1-bib.sdu.dk:2048/summary.do?product=WOS&doc=1&qid=84&SID=Q2eCMF7If3JniHh6eF2&search_mode=GeneralSearch) |
| # 83 | Topic=(empirical study)  Databases=SCI-EXPANDED, SSCI, A&HCI, CPCI-S, CPCI-SSH, BKCI-S, BKCI-SSH Timespan=All Years  Lemmatization=On | [128421](http://apps.webofknowledge.com.proxy1-bib.sdu.dk:2048/summary.do?product=WOS&doc=1&qid=83&SID=Q2eCMF7If3JniHh6eF2&search_mode=GeneralSearch) |
| # 82 | Topic=(focus groups)  Databases=SCI-EXPANDED, SSCI, A&HCI, CPCI-S, CPCI-SSH, BKCI-S, BKCI-SSH Timespan=All Years  Lemmatization=On | [111529](http://apps.webofknowledge.com.proxy1-bib.sdu.dk:2048/summary.do?product=WOS&doc=1&qid=82&SID=Q2eCMF7If3JniHh6eF2&search_mode=GeneralSearch) |
| # 81 | Topic=(focus group)  Databases=SCI-EXPANDED, SSCI, A&HCI, CPCI-S, CPCI-SSH, BKCI-S, BKCI-SSH Timespan=All Years  Lemmatization=On | [111529](http://apps.webofknowledge.com.proxy1-bib.sdu.dk:2048/summary.do?product=WOS&doc=1&qid=81&SID=Q2eCMF7If3JniHh6eF2&search_mode=GeneralSearch) |
| # 80 | Topic=(metaanalyses)  Databases=SCI-EXPANDED, SSCI, A&HCI, CPCI-S, CPCI-SSH, BKCI-S, BKCI-SSH Timespan=All Years  Lemmatization=On | [1222](http://apps.webofknowledge.com.proxy1-bib.sdu.dk:2048/summary.do?product=WOS&doc=1&qid=80&SID=Q2eCMF7If3JniHh6eF2&search_mode=GeneralSearch) |
| # 79 | Topic=(metaanalysis)  Databases=SCI-EXPANDED, SSCI, A&HCI, CPCI-S, CPCI-SSH, BKCI-S, BKCI-SSH Timespan=All Years  Lemmatization=On | [56068](http://apps.webofknowledge.com.proxy1-bib.sdu.dk:2048/summary.do?product=WOS&doc=1&qid=79&SID=Q2eCMF7If3JniHh6eF2&search_mode=GeneralSearch) |
| # 78 | Topic=(meta-analyses)  Databases=SCI-EXPANDED, SSCI, A&HCI, CPCI-S, CPCI-SSH, BKCI-S, BKCI-SSH Timespan=All Years  Lemmatization=On | [54365](http://apps.webofknowledge.com.proxy1-bib.sdu.dk:2048/summary.do?product=WOS&doc=1&qid=78&SID=Q2eCMF7If3JniHh6eF2&search_mode=GeneralSearch) |
| # 77 | Topic=(meta-analysis)  Databases=SCI-EXPANDED, SSCI, A&HCI, CPCI-S, CPCI-SSH, BKCI-S, BKCI-SSH Timespan=All Years  Lemmatization=On | [54215](http://apps.webofknowledge.com.proxy1-bib.sdu.dk:2048/summary.do?product=WOS&doc=1&qid=77&SID=Q2eCMF7If3JniHh6eF2&search_mode=GeneralSearch) |
| # 76 | Topic=(meta analyses)  Databases=SCI-EXPANDED, SSCI, A&HCI, CPCI-S, CPCI-SSH, BKCI-S, BKCI-SSH Timespan=All Years  Lemmatization=On | [62840](http://apps.webofknowledge.com.proxy1-bib.sdu.dk:2048/summary.do?product=WOS&doc=1&qid=76&SID=Q2eCMF7If3JniHh6eF2&search_mode=GeneralSearch) |
| # 75 | Topic=(meta analysis)  Databases=SCI-EXPANDED, SSCI, A&HCI, CPCI-S, CPCI-SSH, BKCI-S, BKCI-SSH Timespan=All Years  Lemmatization=On | [61184](http://apps.webofknowledge.com.proxy1-bib.sdu.dk:2048/summary.do?product=WOS&doc=1&qid=75&SID=Q2eCMF7If3JniHh6eF2&search_mode=GeneralSearch) |
| # 74 | Topic=(reviews)  Databases=SCI-EXPANDED, SSCI, A&HCI, CPCI-S, CPCI-SSH, BKCI-S, BKCI-SSH Timespan=All Years  Lemmatization=On | [1243619](http://apps.webofknowledge.com.proxy1-bib.sdu.dk:2048/summary.do?product=WOS&doc=1&qid=74&SID=Q2eCMF7If3JniHh6eF2&search_mode=GeneralSearch) |
| # 73 | Topic=(review)  Databases=SCI-EXPANDED, SSCI, A&HCI, CPCI-S, CPCI-SSH, BKCI-S, BKCI-SSH Timespan=All Years  Lemmatization=On | [1243619](http://apps.webofknowledge.com.proxy1-bib.sdu.dk:2048/summary.do?product=WOS&doc=1&qid=73&SID=Q2eCMF7If3JniHh6eF2&search_mode=GeneralSearch) |
| # 72 | Topic=(questionnaires)  Databases=SCI-EXPANDED, SSCI, A&HCI, CPCI-S, CPCI-SSH, BKCI-S, BKCI-SSH Timespan=All Years  Lemmatization=On | [275160](http://apps.webofknowledge.com.proxy1-bib.sdu.dk:2048/summary.do?product=WOS&doc=1&qid=72&SID=Q2eCMF7If3JniHh6eF2&search_mode=GeneralSearch) |
| # 71 | Topic=(questionnaire)  Databases=SCI-EXPANDED, SSCI, A&HCI, CPCI-S, CPCI-SSH, BKCI-S, BKCI-SSH Timespan=All Years  Lemmatization=On | [275160](http://apps.webofknowledge.com.proxy1-bib.sdu.dk:2048/summary.do?product=WOS&doc=1&qid=71&SID=Q2eCMF7If3JniHh6eF2&search_mode=GeneralSearch) |
| # 70 | Topic=(interviews)  Databases=SCI-EXPANDED, SSCI, A&HCI, CPCI-S, CPCI-SSH, BKCI-S, BKCI-SSH Timespan=All Years  Lemmatization=On | [232898](http://apps.webofknowledge.com.proxy1-bib.sdu.dk:2048/summary.do?product=WOS&doc=1&qid=70&SID=Q2eCMF7If3JniHh6eF2&search_mode=GeneralSearch) |
| # 69 | Topic=(interview)  Databases=SCI-EXPANDED, SSCI, A&HCI, CPCI-S, CPCI-SSH, BKCI-S, BKCI-SSH Timespan=All Years  Lemmatization=On | [232898](http://apps.webofknowledge.com.proxy1-bib.sdu.dk:2048/summary.do?product=WOS&doc=1&qid=69&SID=Q2eCMF7If3JniHh6eF2&search_mode=GeneralSearch) |
| # 68 | #67 OR #66 OR #65 OR #64  Databases=SCI-EXPANDED, SSCI, A&HCI, CPCI-S, CPCI-SSH, BKCI-S, BKCI-SSH Timespan=All Years  Lemmatization=On | [463072](http://apps.webofknowledge.com.proxy1-bib.sdu.dk:2048/summary.do?product=WOS&doc=1&qid=68&SID=Q2eCMF7If3JniHh6eF2&search_mode=CombineSearches) |
| # 67 | Topic=(hospital settings)  Databases=SCI-EXPANDED, SSCI, A&HCI, CPCI-S, CPCI-SSH, BKCI-S, BKCI-SSH Timespan=All Years  Lemmatization=On | [71061](http://apps.webofknowledge.com.proxy1-bib.sdu.dk:2048/summary.do?product=WOS&doc=1&qid=67&SID=Q2eCMF7If3JniHh6eF2&search_mode=GeneralSearch) |
| # 66 | Topic=(hospital setting)  Databases=SCI-EXPANDED, SSCI, A&HCI, CPCI-S, CPCI-SSH, BKCI-S, BKCI-SSH Timespan=All Years  Lemmatization=On | [79990](http://apps.webofknowledge.com.proxy1-bib.sdu.dk:2048/summary.do?product=WOS&doc=1&qid=66&SID=Q2eCMF7If3JniHh6eF2&search_mode=GeneralSearch) |
| # 65 | Topic=(hospitals)  Databases=SCI-EXPANDED, SSCI, A&HCI, CPCI-S, CPCI-SSH, BKCI-S, BKCI-SSH Timespan=All Years  Lemmatization=On | [463072](http://apps.webofknowledge.com.proxy1-bib.sdu.dk:2048/summary.do?product=WOS&doc=1&qid=65&SID=Q2eCMF7If3JniHh6eF2&search_mode=GeneralSearch) |
| # 64 | Topic=(hospital)  Databases=SCI-EXPANDED, SSCI, A&HCI, CPCI-S, CPCI-SSH, BKCI-S, BKCI-SSH Timespan=All Years  Lemmatization=On | [463072](http://apps.webofknowledge.com.proxy1-bib.sdu.dk:2048/summary.do?product=WOS&doc=1&qid=64&SID=Q2eCMF7If3JniHh6eF2&search_mode=GeneralSearch) |
| # 63 | #62 OR #61 OR #60 OR #59 OR #58 OR #57 OR #56 OR #55 OR #54 OR #53 OR #52 OR #51 OR #50 OR #49 OR #48 OR #47 OR #46 OR #45 OR #44 OR #43 OR #42 OR #41 OR #40 OR #39 OR #38 OR #37 OR #36 OR #35 OR #34 OR #33 OR #32 OR #31  Databases=SCI-EXPANDED, SSCI, A&HCI, CPCI-S, CPCI-SSH, BKCI-S, BKCI-SSH Timespan=All Years  Lemmatization=On | [545141](http://apps.webofknowledge.com.proxy1-bib.sdu.dk:2048/summary.do?product=WOS&doc=1&qid=63&SID=Q2eCMF7If3JniHh6eF2&search_mode=CombineSearches) |
| # 62 | Topic=(decisionmaking)  Databases=SCI-EXPANDED, SSCI, A&HCI, CPCI-S, CPCI-SSH, BKCI-S, BKCI-SSH Timespan=All Years  Lemmatization=On | [2165](http://apps.webofknowledge.com.proxy1-bib.sdu.dk:2048/summary.do?product=WOS&doc=1&qid=62&SID=Q2eCMF7If3JniHh6eF2&search_mode=GeneralSearch) |
| # 61 | Topic=("decision making")  Databases=SCI-EXPANDED, SSCI, A&HCI, CPCI-S, CPCI-SSH, BKCI-S, BKCI-SSH Timespan=All Years  Lemmatization=On | [148228](http://apps.webofknowledge.com.proxy1-bib.sdu.dk:2048/summary.do?product=WOS&doc=1&qid=61&SID=Q2eCMF7If3JniHh6eF2&search_mode=GeneralSearch) |
| # 60 | Topic=("priority setting")  Databases=SCI-EXPANDED, SSCI, A&HCI, CPCI-S, CPCI-SSH, BKCI-S, BKCI-SSH Timespan=All Years  Lemmatization=On | [1497](http://apps.webofknowledge.com.proxy1-bib.sdu.dk:2048/summary.do?product=WOS&doc=1&qid=60&SID=Q2eCMF7If3JniHh6eF2&search_mode=GeneralSearch) |
| # 59 | Topic=(prioritisations)  Databases=SCI-EXPANDED, SSCI, A&HCI, CPCI-S, CPCI-SSH, BKCI-S, BKCI-SSH Timespan=All Years  Lemmatization=On | [5686](http://apps.webofknowledge.com.proxy1-bib.sdu.dk:2048/summary.do?product=WOS&doc=1&qid=59&SID=Q2eCMF7If3JniHh6eF2&search_mode=GeneralSearch) |
| # 58 | Topic=(prioritisation)  Databases=SCI-EXPANDED, SSCI, A&HCI, CPCI-S, CPCI-SSH, BKCI-S, BKCI-SSH Timespan=All Years  Lemmatization=On | [6797](http://apps.webofknowledge.com.proxy1-bib.sdu.dk:2048/summary.do?product=WOS&doc=1&qid=58&SID=Q2eCMF7If3JniHh6eF2&search_mode=GeneralSearch) |
| # 57 | Topic=(prioritizations)  Databases=SCI-EXPANDED, SSCI, A&HCI, CPCI-S, CPCI-SSH, BKCI-S, BKCI-SSH Timespan=All Years  Lemmatization=On | [5686](http://apps.webofknowledge.com.proxy1-bib.sdu.dk:2048/summary.do?product=WOS&doc=1&qid=57&SID=Q2eCMF7If3JniHh6eF2&search_mode=GeneralSearch) |
| # 56 | Topic=(prioritization)  Databases=SCI-EXPANDED, SSCI, A&HCI, CPCI-S, CPCI-SSH, BKCI-S, BKCI-SSH Timespan=All Years  Lemmatization=On | [6797](http://apps.webofknowledge.com.proxy1-bib.sdu.dk:2048/summary.do?product=WOS&doc=1&qid=56&SID=Q2eCMF7If3JniHh6eF2&search_mode=GeneralSearch) |
| # 55 | Topic=(informational needs)  Databases=SCI-EXPANDED, SSCI, A&HCI, CPCI-S, CPCI-SSH, BKCI-S, BKCI-SSH Timespan=All Years  Lemmatization=On | [1699](http://apps.webofknowledge.com.proxy1-bib.sdu.dk:2048/summary.do?product=WOS&doc=1&qid=55&SID=Q2eCMF7If3JniHh6eF2&search_mode=GeneralSearch) |
| # 54 | Topic=(informational need)  Databases=SCI-EXPANDED, SSCI, A&HCI, CPCI-S, CPCI-SSH, BKCI-S, BKCI-SSH Timespan=All Years  Lemmatization=On | [1699](http://apps.webofknowledge.com.proxy1-bib.sdu.dk:2048/summary.do?product=WOS&doc=1&qid=54&SID=Q2eCMF7If3JniHh6eF2&search_mode=GeneralSearch) |
| # 53 | Topic=(information needs)  Databases=SCI-EXPANDED, SSCI, A&HCI, CPCI-S, CPCI-SSH, BKCI-S, BKCI-SSH Timespan=All Years  Lemmatization=On | [170419](http://apps.webofknowledge.com.proxy1-bib.sdu.dk:2048/summary.do?product=WOS&doc=1&qid=53&SID=Q2eCMF7If3JniHh6eF2&search_mode=GeneralSearch) |
| # 52 | Topic=(information need)  Databases=SCI-EXPANDED, SSCI, A&HCI, CPCI-S, CPCI-SSH, BKCI-S, BKCI-SSH Timespan=All Years  Lemmatization=On | [170419](http://apps.webofknowledge.com.proxy1-bib.sdu.dk:2048/summary.do?product=WOS&doc=1&qid=52&SID=Q2eCMF7If3JniHh6eF2&search_mode=GeneralSearch) |
| # 51 | Topic=(decision inputs)  Databases=SCI-EXPANDED, SSCI, A&HCI, CPCI-S, CPCI-SSH, BKCI-S, BKCI-SSH Timespan=All Years  Lemmatization=On | [16542](http://apps.webofknowledge.com.proxy1-bib.sdu.dk:2048/summary.do?product=WOS&doc=1&qid=51&SID=Q2eCMF7If3JniHh6eF2&search_mode=GeneralSearch) |
| # 50 | Topic=(decision input)  Databases=SCI-EXPANDED, SSCI, A&HCI, CPCI-S, CPCI-SSH, BKCI-S, BKCI-SSH Timespan=All Years  Lemmatization=On | [16542](http://apps.webofknowledge.com.proxy1-bib.sdu.dk:2048/summary.do?product=WOS&doc=1&qid=50&SID=Q2eCMF7If3JniHh6eF2&search_mode=GeneralSearch) |
| # 49 | Topic=(technology adoptions)  Databases=SCI-EXPANDED, SSCI, A&HCI, CPCI-S, CPCI-SSH, BKCI-S, BKCI-SSH Timespan=All Years  Lemmatization=On | [15069](http://apps.webofknowledge.com.proxy1-bib.sdu.dk:2048/summary.do?product=WOS&doc=1&qid=49&SID=Q2eCMF7If3JniHh6eF2&search_mode=GeneralSearch) |
| # 48 | Topic=(technology adoption)  Databases=SCI-EXPANDED, SSCI, A&HCI, CPCI-S, CPCI-SSH, BKCI-S, BKCI-SSH Timespan=All Years  Lemmatization=On | [15069](http://apps.webofknowledge.com.proxy1-bib.sdu.dk:2048/summary.do?product=WOS&doc=1&qid=48&SID=Q2eCMF7If3JniHh6eF2&search_mode=GeneralSearch) |
| # 47 | Topic=(economic analyses)  Databases=SCI-EXPANDED, SSCI, A&HCI, CPCI-S, CPCI-SSH, BKCI-S, BKCI-SSH Timespan=All Years  Lemmatization=On | [119568](http://apps.webofknowledge.com.proxy1-bib.sdu.dk:2048/summary.do?product=WOS&doc=1&qid=47&SID=Q2eCMF7If3JniHh6eF2&search_mode=GeneralSearch) |
| # 46 | Topic=(economic analysis)  Databases=SCI-EXPANDED, SSCI, A&HCI, CPCI-S, CPCI-SSH, BKCI-S, BKCI-SSH Timespan=All Years  Lemmatization=On | [98580](http://apps.webofknowledge.com.proxy1-bib.sdu.dk:2048/summary.do?product=WOS&doc=1&qid=46&SID=Q2eCMF7If3JniHh6eF2&search_mode=GeneralSearch) |
| # 45 | Topic=(economic evaluations)  Databases=SCI-EXPANDED, SSCI, A&HCI, CPCI-S, CPCI-SSH, BKCI-S, BKCI-SSH Timespan=All Years  Lemmatization=On | [27879](http://apps.webofknowledge.com.proxy1-bib.sdu.dk:2048/summary.do?product=WOS&doc=1&qid=45&SID=Q2eCMF7If3JniHh6eF2&search_mode=GeneralSearch) |
| # 44 | Topic=(economic evaluation)  Databases=SCI-EXPANDED, SSCI, A&HCI, CPCI-S, CPCI-SSH, BKCI-S, BKCI-SSH Timespan=All Years  Lemmatization=On | [27879](http://apps.webofknowledge.com.proxy1-bib.sdu.dk:2048/summary.do?product=WOS&doc=1&qid=44&SID=Q2eCMF7If3JniHh6eF2&search_mode=GeneralSearch) |
| # 43 | Topic=(mini-HTA's)  Databases=SCI-EXPANDED, SSCI, A&HCI, CPCI-S, CPCI-SSH, BKCI-S, BKCI-SSH Timespan=All Years  Lemmatization=On | 0 |
| # 42 | Topic=(mini-HTAs)  Databases=SCI-EXPANDED, SSCI, A&HCI, CPCI-S, CPCI-SSH, BKCI-S, BKCI-SSH Timespan=All Years  Lemmatization=On | [2](http://apps.webofknowledge.com.proxy1-bib.sdu.dk:2048/summary.do?product=WOS&doc=1&qid=42&SID=Q2eCMF7If3JniHh6eF2&search_mode=GeneralSearch) |
| # 41 | Topic=(mini-HTA)  Databases=SCI-EXPANDED, SSCI, A&HCI, CPCI-S, CPCI-SSH, BKCI-S, BKCI-SSH Timespan=All Years  Lemmatization=On | [8](http://apps.webofknowledge.com.proxy1-bib.sdu.dk:2048/summary.do?product=WOS&doc=1&qid=41&SID=Q2eCMF7If3JniHh6eF2&search_mode=GeneralSearch) |
| # 40 | Topic=(mini HTA's)  Databases=SCI-EXPANDED, SSCI, A&HCI, CPCI-S, CPCI-SSH, BKCI-S, BKCI-SSH Timespan=All Years  Lemmatization=On | 0 |
| # 39 | Topic=(mini HTAs)  Databases=SCI-EXPANDED, SSCI, A&HCI, CPCI-S, CPCI-SSH, BKCI-S, BKCI-SSH Timespan=All Years  Lemmatization=On | [2](http://apps.webofknowledge.com.proxy1-bib.sdu.dk:2048/summary.do?product=WOS&doc=1&qid=39&SID=Q2eCMF7If3JniHh6eF2&search_mode=GeneralSearch) |
| # 38 | Topic=(mini HTA)  Databases=SCI-EXPANDED, SSCI, A&HCI, CPCI-S, CPCI-SSH, BKCI-S, BKCI-SSH Timespan=All Years  Lemmatization=On | [11](http://apps.webofknowledge.com.proxy1-bib.sdu.dk:2048/summary.do?product=WOS&doc=1&qid=38&SID=Q2eCMF7If3JniHh6eF2&search_mode=GeneralSearch) |
| # 37 | Topic=(HTA's)  Databases=SCI-EXPANDED, SSCI, A&HCI, CPCI-S, CPCI-SSH, BKCI-S, BKCI-SSH Timespan=All Years  Lemmatization=On | [6](http://apps.webofknowledge.com.proxy1-bib.sdu.dk:2048/summary.do?product=WOS&doc=1&qid=37&SID=Q2eCMF7If3JniHh6eF2&search_mode=GeneralSearch) |
| # 36 | Topic=(HTAs)  Databases=SCI-EXPANDED, SSCI, A&HCI, CPCI-S, CPCI-SSH, BKCI-S, BKCI-SSH Timespan=All Years  Lemmatization=On | [130](http://apps.webofknowledge.com.proxy1-bib.sdu.dk:2048/summary.do?product=WOS&doc=1&qid=36&SID=Q2eCMF7If3JniHh6eF2&search_mode=GeneralSearch) |
| # 35 | Topic=(HTA)  Databases=SCI-EXPANDED, SSCI, A&HCI, CPCI-S, CPCI-SSH, BKCI-S, BKCI-SSH Timespan=All Years  Lemmatization=On | [1361](http://apps.webofknowledge.com.proxy1-bib.sdu.dk:2048/summary.do?product=WOS&doc=1&qid=35&SID=Q2eCMF7If3JniHh6eF2&search_mode=GeneralSearch) |
| # 34 | Topic=(health technology assessments)  Databases=SCI-EXPANDED, SSCI, A&HCI, CPCI-S, CPCI-SSH, BKCI-S, BKCI-SSH Timespan=All Years  Lemmatization=On | [5759](http://apps.webofknowledge.com.proxy1-bib.sdu.dk:2048/summary.do?product=WOS&doc=1&qid=34&SID=Q2eCMF7If3JniHh6eF2&search_mode=GeneralSearch) |
| # 33 | Topic=(health technology assessment)  Databases=SCI-EXPANDED, SSCI, A&HCI, CPCI-S, CPCI-SSH, BKCI-S, BKCI-SSH Timespan=All Years  Lemmatization=On | [5759](http://apps.webofknowledge.com.proxy1-bib.sdu.dk:2048/summary.do?product=WOS&doc=1&qid=33&SID=Q2eCMF7If3JniHh6eF2&search_mode=GeneralSearch) |
| # 32 | Topic=(program evaluations)  Databases=SCI-EXPANDED, SSCI, A&HCI, CPCI-S, CPCI-SSH, BKCI-S, BKCI-SSH Timespan=All Years  Lemmatization=On | [81694](http://apps.webofknowledge.com.proxy1-bib.sdu.dk:2048/summary.do?product=WOS&doc=1&qid=32&SID=Q2eCMF7If3JniHh6eF2&search_mode=GeneralSearch) |
| # 31 | Topic=(program evaluation)  Databases=SCI-EXPANDED, SSCI, A&HCI, CPCI-S, CPCI-SSH, BKCI-S, BKCI-SSH Timespan=All Years  Lemmatization=On | [81694](http://apps.webofknowledge.com.proxy1-bib.sdu.dk:2048/summary.do?product=WOS&doc=1&qid=31&SID=Q2eCMF7If3JniHh6eF2&search_mode=GeneralSearch) |
| # 30 | #29 OR #28 OR #27 OR #26 OR #25 OR #24 OR #23 OR #22 OR #21 OR #20 OR #19 OR #18 OR #17 OR #16 OR #15 OR #14 OR #13 OR #12 OR #11 OR #10 OR #9 OR #8 OR #7 OR #6 OR #5 OR #4 OR #3 OR #2 OR #1  Databases=SCI-EXPANDED, SSCI, A&HCI, CPCI-S, CPCI-SSH, BKCI-S, BKCI-SSH Timespan=All Years  Lemmatization=On | [51104](http://apps.webofknowledge.com.proxy1-bib.sdu.dk:2048/summary.do?product=WOS&doc=1&qid=30&SID=Q2eCMF7If3JniHh6eF2&search_mode=CombineSearches) |
| # 29 | Topic=("healthcare stakeholders")  Databases=SCI-EXPANDED, SSCI, A&HCI, CPCI-S, CPCI-SSH, BKCI-S, BKCI-SSH Timespan=All Years  Lemmatization=On | [33](http://apps.webofknowledge.com.proxy1-bib.sdu.dk:2048/summary.do?product=WOS&doc=1&qid=29&SID=Q2eCMF7If3JniHh6eF2&search_mode=GeneralSearch) |
| # 28 | Topic=("healthcare stakeholder")  Databases=SCI-EXPANDED, SSCI, A&HCI, CPCI-S, CPCI-SSH, BKCI-S, BKCI-SSH Timespan=All Years  Lemmatization=On | [6](http://apps.webofknowledge.com.proxy1-bib.sdu.dk:2048/summary.do?product=WOS&doc=1&qid=28&SID=Q2eCMF7If3JniHh6eF2&search_mode=GeneralSearch) |
| # 27 | Topic=("health care stakeholders")  Databases=SCI-EXPANDED, SSCI, A&HCI, CPCI-S, CPCI-SSH, BKCI-S, BKCI-SSH Timespan=All Years  Lemmatization=On | [41](http://apps.webofknowledge.com.proxy1-bib.sdu.dk:2048/summary.do?product=WOS&doc=1&qid=27&SID=Q2eCMF7If3JniHh6eF2&search_mode=GeneralSearch) |
| # 26 | Topic=("health care stakeholder")  Databases=SCI-EXPANDED, SSCI, A&HCI, CPCI-S, CPCI-SSH, BKCI-S, BKCI-SSH Timespan=All Years  Lemmatization=On | [5](http://apps.webofknowledge.com.proxy1-bib.sdu.dk:2048/summary.do?product=WOS&doc=1&qid=26&SID=Q2eCMF7If3JniHh6eF2&search_mode=GeneralSearch) |
| # 25 | Topic=(policymakers)  Databases=SCI-EXPANDED, SSCI, A&HCI, CPCI-S, CPCI-SSH, BKCI-S, BKCI-SSH Timespan=All Years  Lemmatization=On | [7463](http://apps.webofknowledge.com.proxy1-bib.sdu.dk:2048/summary.do?product=WOS&doc=1&qid=25&SID=Q2eCMF7If3JniHh6eF2&search_mode=GeneralSearch) |
| # 24 | Topic=(policymaker)  Databases=SCI-EXPANDED, SSCI, A&HCI, CPCI-S, CPCI-SSH, BKCI-S, BKCI-SSH Timespan=All Years  Lemmatization=On | [7463](http://apps.webofknowledge.com.proxy1-bib.sdu.dk:2048/summary.do?product=WOS&doc=1&qid=24&SID=Q2eCMF7If3JniHh6eF2&search_mode=GeneralSearch) |
| # 23 | Topic=("policy makers")  Databases=SCI-EXPANDED, SSCI, A&HCI, CPCI-S, CPCI-SSH, BKCI-S, BKCI-SSH Timespan=All Years  Lemmatization=On | [18714](http://apps.webofknowledge.com.proxy1-bib.sdu.dk:2048/summary.do?product=WOS&doc=1&qid=23&SID=Q2eCMF7If3JniHh6eF2&search_mode=GeneralSearch) |
| # 22 | Topic=("policy maker")  Databases=SCI-EXPANDED, SSCI, A&HCI, CPCI-S, CPCI-SSH, BKCI-S, BKCI-SSH Timespan=All Years  Lemmatization=On | [609](http://apps.webofknowledge.com.proxy1-bib.sdu.dk:2048/summary.do?product=WOS&doc=1&qid=22&SID=Q2eCMF7If3JniHh6eF2&search_mode=GeneralSearch) |
| # 21 | Topic=(decisionmakers)  Databases=SCI-EXPANDED, SSCI, A&HCI, CPCI-S, CPCI-SSH, BKCI-S, BKCI-SSH Timespan=All Years  Lemmatization=On | [574](http://apps.webofknowledge.com.proxy1-bib.sdu.dk:2048/summary.do?product=WOS&doc=1&qid=21&SID=Q2eCMF7If3JniHh6eF2&search_mode=GeneralSearch) |
| # 20 | Topic=(decisionmaker)  Databases=SCI-EXPANDED, SSCI, A&HCI, CPCI-S, CPCI-SSH, BKCI-S, BKCI-SSH Timespan=All Years  Lemmatization=On | [574](http://apps.webofknowledge.com.proxy1-bib.sdu.dk:2048/summary.do?product=WOS&doc=1&qid=20&SID=Q2eCMF7If3JniHh6eF2&search_mode=GeneralSearch) |
| # 19 | Topic=("decision makers")  Databases=SCI-EXPANDED, SSCI, A&HCI, CPCI-S, CPCI-SSH, BKCI-S, BKCI-SSH Timespan=All Years  Lemmatization=On | [17477](http://apps.webofknowledge.com.proxy1-bib.sdu.dk:2048/summary.do?product=WOS&doc=1&qid=19&SID=Q2eCMF7If3JniHh6eF2&search_mode=GeneralSearch) |
| # 18 | Topic=("decision maker")  Databases=SCI-EXPANDED, SSCI, A&HCI, CPCI-S, CPCI-SSH, BKCI-S, BKCI-SSH Timespan=All Years  Lemmatization=On | [7016](http://apps.webofknowledge.com.proxy1-bib.sdu.dk:2048/summary.do?product=WOS&doc=1&qid=18&SID=Q2eCMF7If3JniHh6eF2&search_mode=GeneralSearch) |
| # 17 | Topic=("clinical administrative managers")  Databases=SCI-EXPANDED, SSCI, A&HCI, CPCI-S, CPCI-SSH, BKCI-S, BKCI-SSH Timespan=All Years  Lemmatization=On | 0 |
| # 16 | Topic=("clinical administrative manager")  Databases=SCI-EXPANDED, SSCI, A&HCI, CPCI-S, CPCI-SSH, BKCI-S, BKCI-SSH Timespan=All Years  Lemmatization=On | 0 |
| # 15 | Topic=("clinical managers")  Databases=SCI-EXPANDED, SSCI, A&HCI, CPCI-S, CPCI-SSH, BKCI-S, BKCI-SSH Timespan=All Years  Lemmatization=On | [47](http://apps.webofknowledge.com.proxy1-bib.sdu.dk:2048/summary.do?product=WOS&doc=1&qid=15&SID=Q2eCMF7If3JniHh6eF2&search_mode=GeneralSearch) |
| # 14 | Topic=("clinical manager")  Databases=SCI-EXPANDED, SSCI, A&HCI, CPCI-S, CPCI-SSH, BKCI-S, BKCI-SSH Timespan=All Years  Lemmatization=On | [20](http://apps.webofknowledge.com.proxy1-bib.sdu.dk:2048/summary.do?product=WOS&doc=1&qid=14&SID=Q2eCMF7If3JniHh6eF2&search_mode=GeneralSearch) |
| # 13 | Topic=("hospital leaders")  Databases=SCI-EXPANDED, SSCI, A&HCI, CPCI-S, CPCI-SSH, BKCI-S, BKCI-SSH Timespan=All Years  Lemmatization=On | [77](http://apps.webofknowledge.com.proxy1-bib.sdu.dk:2048/summary.do?product=WOS&doc=1&qid=13&SID=Q2eCMF7If3JniHh6eF2&search_mode=GeneralSearch) |
| # 12 | Topic=("hospital leader")  Databases=SCI-EXPANDED, SSCI, A&HCI, CPCI-S, CPCI-SSH, BKCI-S, BKCI-SSH Timespan=All Years  Lemmatization=On | [1](http://apps.webofknowledge.com.proxy1-bib.sdu.dk:2048/summary.do?product=WOS&doc=1&qid=12&SID=Q2eCMF7If3JniHh6eF2&search_mode=GeneralSearch) |
| # 11 | Topic=("hospital directors")  Databases=SCI-EXPANDED, SSCI, A&HCI, CPCI-S, CPCI-SSH, BKCI-S, BKCI-SSH Timespan=All Years  Lemmatization=On | [46](http://apps.webofknowledge.com.proxy1-bib.sdu.dk:2048/summary.do?product=WOS&doc=1&qid=11&SID=Q2eCMF7If3JniHh6eF2&search_mode=GeneralSearch) |
| # 10 | Topic=("hospital director")  Databases=SCI-EXPANDED, SSCI, A&HCI, CPCI-S, CPCI-SSH, BKCI-S, BKCI-SSH Timespan=All Years  Lemmatization=On | [20](http://apps.webofknowledge.com.proxy1-bib.sdu.dk:2048/summary.do?product=WOS&doc=1&qid=10&SID=Q2eCMF7If3JniHh6eF2&search_mode=GeneralSearch) |
| # 9 | Topic=("hospital managers")  Databases=SCI-EXPANDED, SSCI, A&HCI, CPCI-S, CPCI-SSH, BKCI-S, BKCI-SSH Timespan=All Years  Lemmatization=On | [286](http://apps.webofknowledge.com.proxy1-bib.sdu.dk:2048/summary.do?product=WOS&doc=1&qid=9&SID=Q2eCMF7If3JniHh6eF2&search_mode=GeneralSearch) |
| # 8 | Topic=("hospital manager")  Databases=SCI-EXPANDED, SSCI, A&HCI, CPCI-S, CPCI-SSH, BKCI-S, BKCI-SSH Timespan=All Years  Lemmatization=On | [18](http://apps.webofknowledge.com.proxy1-bib.sdu.dk:2048/summary.do?product=WOS&doc=1&qid=8&SID=Q2eCMF7If3JniHh6eF2&search_mode=GeneralSearch) |
| # 7 | Topic=("hospital chief executive officers")  Databases=SCI-EXPANDED, SSCI, A&HCI, CPCI-S, CPCI-SSH, BKCI-S, BKCI-SSH Timespan=All Years  Lemmatization=On | [15](http://apps.webofknowledge.com.proxy1-bib.sdu.dk:2048/summary.do?product=WOS&doc=1&qid=7&SID=Q2eCMF7If3JniHh6eF2&search_mode=GeneralSearch) |
| # 6 | Topic=("hospital chief executive officer")  Databases=SCI-EXPANDED, SSCI, A&HCI, CPCI-S, CPCI-SSH, BKCI-S, BKCI-SSH Timespan=All Years  Lemmatization=On | [3](http://apps.webofknowledge.com.proxy1-bib.sdu.dk:2048/summary.do?product=WOS&doc=1&qid=6&SID=Q2eCMF7If3JniHh6eF2&search_mode=GeneralSearch) |
| # 5 | Topic=("hospital CEO's")  Databases=SCI-EXPANDED, SSCI, A&HCI, CPCI-S, CPCI-SSH, BKCI-S, BKCI-SSH Timespan=All Years  Lemmatization=On | 0 |
| # 4 | Topic=("hospital CEOs")  Databases=SCI-EXPANDED, SSCI, A&HCI, CPCI-S, CPCI-SSH, BKCI-S, BKCI-SSH Timespan=All Years  Lemmatization=On | [46](http://apps.webofknowledge.com.proxy1-bib.sdu.dk:2048/summary.do?product=WOS&doc=1&qid=4&SID=Q2eCMF7If3JniHh6eF2&search_mode=GeneralSearch) |
| # 3 | Topic=("hospital CEO")  Databases=SCI-EXPANDED, SSCI, A&HCI, CPCI-S, CPCI-SSH, BKCI-S, BKCI-SSH Timespan=All Years  Lemmatization=On | [17](http://apps.webofknowledge.com.proxy1-bib.sdu.dk:2048/summary.do?product=WOS&doc=1&qid=3&SID=Q2eCMF7If3JniHh6eF2&search_mode=GeneralSearch) |
| # 2 | Topic=("hospital administrators")  Databases=SCI-EXPANDED, SSCI, A&HCI, CPCI-S, CPCI-SSH, BKCI-S, BKCI-SSH Timespan=All Years  Lemmatization=On | [553](http://apps.webofknowledge.com.proxy1-bib.sdu.dk:2048/summary.do?product=WOS&doc=1&qid=2&SID=Q2eCMF7If3JniHh6eF2&search_mode=GeneralSearch) |
| # 1 | Topic=("hospital administrator")  Databases=SCI-EXPANDED, SSCI, A&HCI, CPCI-S, CPCI-SSH, BKCI-S, BKCI-SSH Timespan=All Years  Lemmatization=On | [87](http://apps.webofknowledge.com.proxy1-bib.sdu.dk:2048/summary.do?product=WOS&doc=1&qid=1&SID=Q2eCMF7If3JniHh6eF2&search_mode=GeneralSearch) |
